# Inhibition of fatty acid uptake by TGR5 prevents diabetic cardiomyopathy

**DOI:** 10.1038/s42255-024-01036-5

**Published:** 2024-05-02

**Authors:** Hu Wang, Jiaxing Wang, Hao Cui, Chenyu Fan, Yuzhou Xue, Huiying Liu, Hui Li, Jianping Li, Houhua Li, Ying Sun, Wengong Wang, Jiangping Song, Changtao Jiang, Ming Xu

**Affiliations:** 1 Department of Cardiology and Institute of Vascular Medicine, Peking University Third Hospital, State Key Laboratory of Vascular Homeostasis and Remodeling, NHC Key Laboratory of Cardiovascular Molecular Biology and Regulatory Peptides, Beijing Key Laboratory of Cardiovascular Receptors Research, Peking University, Beijing, China; 2 State Key Laboratory of Cardiovascular Disease, Fuwai Hospital, National Center for Cardiovascular Diseases, Chinese Academy of Medical Sciences and Peking Union Medical College (CAMS & PUMC), Beijing, China; 3 Department of Physiology and Pathophysiology, School of Basic Medical Sciences, State Key Laboratory of Vascular Homeostasis and Remodeling, Peking University, Beijing, China; 4 Department of Cardiology, Peking University First Hospital, State Key Laboratory of Vascular Homeostasis and Remodeling, Peking University, Beijing, China; 5 State Key Laboratory of Natural and Biomimetic Drugs, Chemical Biology Center, School of Pharmaceutical Sciences, Peking University, Beijing, China; 6 Jiangsu Key Laboratory of New Drug Research and Clinical Pharmacy, Xuzhou Medical University, Xuzhou, China; 7 Department of Biochemistry and Molecular Biology, Beijing Key Laboratory of Protein Posttranslational Modifications and Cell Function, School of Basic Medical Sciences, Peking University Health Science Center, Beijing, China; 8 Center of Basic Medical Research, Institute of Medical Innovation and Research, Peking University Third Hospital, Beijing, China; 9 Research Unit of Medical Science Research Management/Basic and Clinical Research of Metabolic Cardiovascular Diseases, Chinese Academy of Medical Sciences, Beijing, China

**Keywords:** Metabolic diseases, Heart failure, Preclinical research, Heart failure

## Abstract

Diabetic cardiomyopathy is characterized by myocardial lipid accumulation and cardiac dysfunction. Bile acid metabolism is known to play a crucial role in cardiovascular and metabolic diseases. Takeda G-protein-coupled receptor 5 (TGR5), a major bile acid receptor, has been implicated in metabolic regulation and myocardial protection. However, the precise involvement of the bile acid–TGR5 pathway in maintaining cardiometabolic homeostasis remains unclear. Here we show decreased plasma bile acid levels in both male and female participants with diabetic myocardial injury. Additionally, we observe increased myocardial lipid accumulation and cardiac dysfunction in cardiomyocyte-specific TGR5-deleted mice (both male and female) subjected to a high-fat diet and streptozotocin treatment or bred on the diabetic *db/db* genetic background. Further investigation reveals that TGR5 deletion enhances cardiac fatty acid uptake, resulting in lipid accumulation. Mechanistically, TGR5 deletion promotes localization of CD36 on the plasma membrane through the upregulation of CD36 palmitoylation mediated by the palmitoyl acyltransferase DHHC4. Our findings indicate that the TGR5–DHHC4 pathway regulates cardiac fatty acid uptake, which highlights the therapeutic potential of targeting TGR5 in the management of diabetic cardiomyopathy.

## Abstract (web summary)

TGR5 activation improves myocardial lipid accumulation and cardiac dysfunction in diabetic mice, offering promise for managing diabetic cardiomyopathy.

## Main

Metabolic imbalances, particularly in lipid metabolism imbalance, are key characteristics of diabetic cardiomyopathy (DbCM), leading to myocardial remodelling and cardiac dysfunction^1,2^. Maintaining metabolic balance in the myocardium is a primary therapeutic strategy for DbCM. Fatty acids serve as the main substrates for ATP production in the heart, accounting for up to 70% of the energy supply in normal adult hearts, and even more so in diabetes mellitus^3^. Fatty acid metabolism encompasses the uptake, synthesis and consumption of fatty acids. In diabetes, insulin resistance and elevated circulating free fatty acids (FFAs) promote fatty acid uptake and trigger intracellular lipid accumulation^2^. The redundant bulk of fatty acids turns to introduce cellular lipotoxicity and disrupts energy supply, contributing significantly to cardiac dysfunction in DbCM^4^. Cluster of differentiation (CD36), a membrane-localized fatty acid translocase, is considered the primary fatty acid transporter and plays a crucial role in lipid homeostasis^5,6^. CD36 assumes the main responsibility for excess cellular fatty acid uptake in diabetes^7^. However, the detailed mechanisms underlying CD36 overactivity, and its regulatory strategies remain incompletely understood.

As the derivatives of cholesterol, bile acids and their metabolism cascades are involved in a variety of metabolic diseases, including atherosclerosis, obesity and type 2 diabetes, through binding to membrane-bound TGR5 (also known as GPBAR1) and nuclear farnesoid X receptors^8–11^. Altered plasma levels of bile acids, particularly those biased towards TGR5 receptor activation, have been observed in individuals with diabetes and animal models of diabetes^12,13^, suggesting an association between diabetes and the bile acid metabolic pathway. TGR5 is expressed in various tissues, including adipose^14,15^, intestine^16,17^, gallbladder^18^ and brain^8,19^, and extensively modulates metabolic homeostasis. Previous studies, including our own, have reported that TGR5 is expressed in the heart and is involved in maintaining cardiac function after infarction and cirrhotic cardiomyopathy^20,21^. Regarding lipid metabolism, TGR5 deletion has been shown to exacerbate alcohol-induced steatosis and liver injury by reducing fatty acid oxidation (FAO) from white adipose tissue and increasing fatty acid absorption into the liver^22^. Considering the abnormal bile acid–TGR5 signalling pathway in diabetes and the close relationship between TGR5 and metabolic homeostasis, and the mysterious relationships between the bile acid–TGR5 signalling pathway and the regulation of CD36, we explored the effect of TGR5 on DbCM and lipid metabolism.

In this study, we investigated the role of TGR5 in cardiac lipid metabolism using cardiomyocyte-specific TGR5-deleted mice subjected to a high-fat diet (HFD)/streptozotocin (STZ) or bred on the diabetic *db/db* genetic background to induce cardiolipotoxicity. Our findings revealed that TGR5 inhibits fatty acid uptake and lipid accumulation in the heart. These results were further validated in mice treated with the TGR5 agonist INT-777 or bile acids deoxycholic acid (DCA) and taurocholic acid (TCA). Furthermore, TGR5 deletion promoted CD36 palmitoylation and membrane localization in cardiomyocytes, a process mediated by the palmitoyl acyltransferase DHHC4. Functional abnormalities induced by TGR5 deletion in cardiomyocytes were reversed after DHHC4 knockdown (KD). Notably, in participants with diabetic myocardial injury, TGR5-biased bile acids, especially DCA, were found to be suppressed. Our data underscore the potential of TGR5 as a therapeutic target for DbCM due to its role in regulating cardiac lipid metabolism.

## Results

### Cardiac-specific TGR5 deletion aggravates cardiac dysfunction

**Fig. 1:**
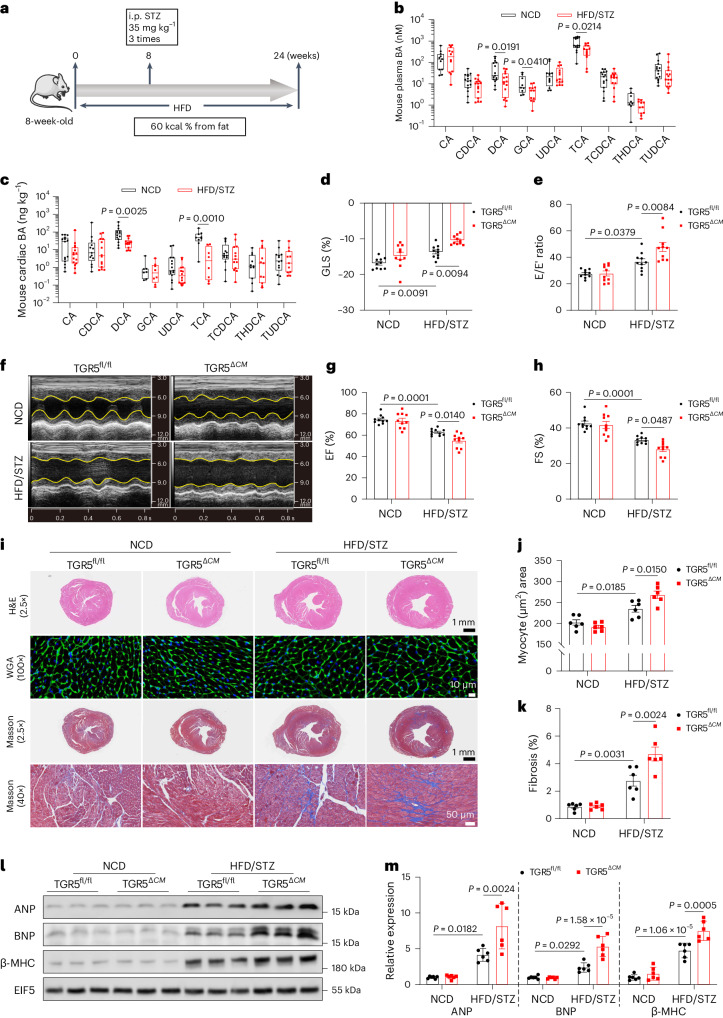
Cardiac-specific deletion of TGR5 exacerbates cardiac dysfunction and remodelling in mice with HFD/STZ-induced DbCM. **a**, Schematic showing the procedure of HFD/STZ-induced diabetic mice. Created with BioRender.com with modifications. **b**,**c**, Plasma (**b**) and cardiac (**c**) bile acid (BA) profiles in mice with a normal control diet (NCD) and HFD/STZ-induced diabetic myocardial injury. *n* = 15. CA, cholic acid; CDCA, chenodeoxycholic acid; GCA, glycocholic acid; UDCA, ursodeoxycholic acid; TCDCA, taurochenodeoxycholic acid; THDCA, taurohyodeoxycholic acid; TUDCA, tauroursodeoxycholic acid. **d**, Left ventricular GLS was calculated using Vevo software. *n* = 10. **e**, Ratio of flow Doppler E wave amplitude to tissue Doppler E′ wave amplitude (E/E′). *n* = 10. **f**, Representative left ventricular M-mode echocardiographic images. **g**, Quantification of left ventricular EF. *n* = 10. **h**, Quantification of left ventricular fractional shortening (FS). *n* = 10. **i**–**k**, Representative images of H&E, WGA and Masson’s trichrome staining of cardiac tissues (**i**). Scale bars, 1 mm for H&E; 10 μm for WGA; 1 mm and 50 μm for Masson’s trichrome staining. Quantification of myocyte area in WGA staining (**j**). *n* = 6. Quantification of cardiac fibrosis area in Masson’s trichrome staining (**k**). *n* = 6. **l**,**m**, Representative western blot images of ANP, BNP and β-MHC in cardiac tissues (**l**). Quantification of western blots shown in **l** (**m**). *n* = 6. For box plots, the midline represents the median; box represents the interquartile range (IQR) between the first and third quartiles; and whiskers represent the lowest or highest values within 1.5 times IQR from the first or third quartiles. Two-tailed non-parametric Mann–Whitney test (**b** and **c**) was used for statistical analysis. The remaining data are presented as the mean ± s.e.m. Statistical significance was evaluated by two-way analysis of variance (ANOVA) followed by Tukey’s post hoc test (**d**, **e**, **g**, **h**, **j**, **k** and **m**). Source data

**Extended Data Fig. 1:**
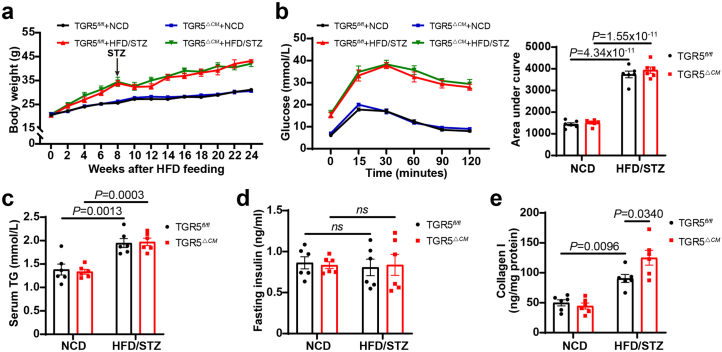
Cardiomyocyte-specific TGR5 deletion does not affect body weight, glycemic or lipidemic control in mice. **a**, Continuous body weight detection of TGR5^*fl/fl*^ and TGR5^*ΔCM*^ mice challenged with HFD/STZ for 24 weeks. n = 6. **b**, Intraperitoneal glucose tolerance test of TGR5^*fl/fl*^ and TGR5^*ΔCM*^ mice challenged with HFD/STZ at 24 weeks (1 g/kg D-glucose). n = 6. **c,d**, Serum TG (**c**) and fasting insulin (**d**) levels of TGR5^*fl/fl*^ and TGR5^*ΔCM*^ mice challenged with HFD/STZ at 24 weeks. n = 6. **e**, Cardiac collagen I levels of TGR5^*fl/fl*^ and TGR5^*ΔCM*^ mice challenged with HFD/STZ at 24 weeks. n = 6. Data are presented as mean ± SEM. Statistical significance was evaluated by two-way ANOVA followed by Tukey’s post-hoc test. Source data

**Extended Data Fig. 2:**
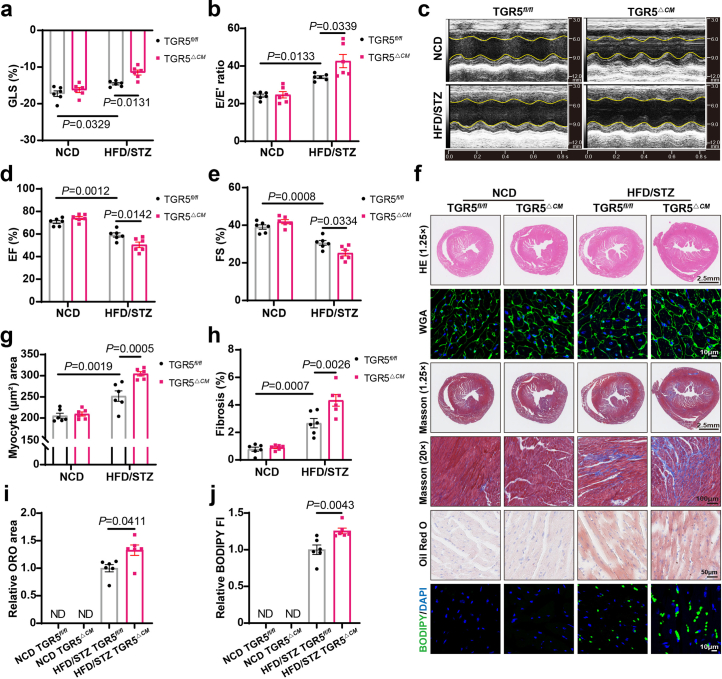
Cardiac-specific TGR5 deletion aggravates cardiac dysfunction and cardiolipotoxicity in female mice with HFD/STZ-induced diabetic cardiomyopathy. **a**, Left ventricular global longitudinal strain (GLS) was calculated using Vevo Software. n = 6. **b**, Ratio of flow Doppler E wave amplitude to tissue Doppler E’ wave amplitude (E/E’). n = 6. **c**, Representative left ventricular M-mode echocardiographic images. **d**, Quantification of left ventricular ejection fraction (EF). n = 6. **e**, Quantification of left ventricular fractional shortening (FS). n = 6. **f**, Representative images of H&E, WGA, Masson’s trichrome, Oil Red O and BODIPY 493/503 staining (green color) images of cardiac tissues from TGR5^*fl/fl*^ and TGR5^*ΔCM*^ mice challenged with HFD/STZ. Scale bar, 2.5 mm for H&E; 10 μm for WGA; 2.5 mm and 100 μm for Masson’s trichrome staining; 50 μm for Oil Red O staining; 10 μm for BODIPY 493/503 staining. The nucleus was stained with DAPI (blue color). **g**, Quantification of myocyte area in WGA staining. n = 6. **h**, Quantification of cardiac fibrosis area in Masson’s trichrome staining. n = 6. **i**, Quantitative analysis of Oil Red O staining. n = 6. **j**, BODIPY intensity by Image J. n = 6. Data are presented as mean ± SEM. Statistical significance was evaluated by two-tailed nonparametric Mann–Whitney test (**i,j**), or two-way ANOVA followed by Tukey’s post-hoc test (**a,b,d,e,g,h**). Source data

**Extended Data Fig. 3:**
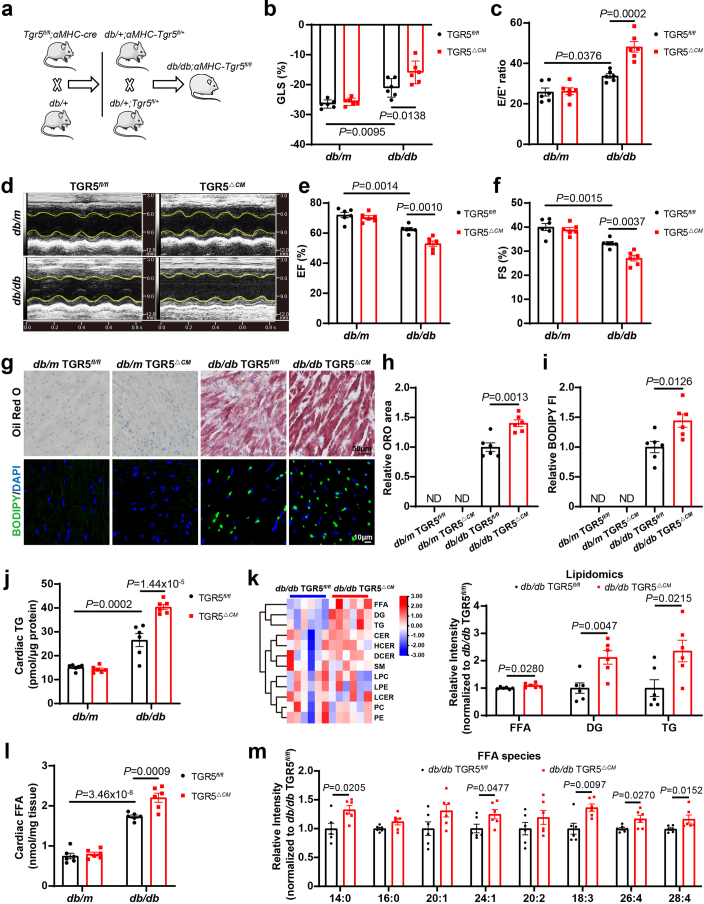
Cardiac-specific TGR5 deletion aggravates cardiac dysfunction and cardiolipotoxicity in *db/db* mice. **a**, Experimental scheme for generating *db/db;αMHC-Tgr5*^*fl/fl*^ mice (*db/db* TGR5^*ΔCM*^). Mice aged 20 weeks were used in the following experiments. Created with BioRender.com with modifications. **b–f**, Left ventricular GLS was calculated by Vevo Software (**b**). n = 6. Ratio of flow Doppler E wave amplitude to tissue Doppler E′ wave amplitude (E/E’) (**c**). n = 6. Representative left ventricular M-mode echocardiographic images (**d**). Quantification of left ventricular EF (**e**). n = 6. Quantification of left ventricular FS (**f**). n = 6. **g–j**, Representative Oil Red O (above) and BODIPY 493/503 staining (below, green color) images of cardiac tissues from *db/db* TGR5^*fl/fl*^ and *db/db* TGR5^*ΔCM*^ mice (**g**). Scale bar, 50 μm for Oil Red O staining; 10 μm for BODIPY 493/503 staining. The nucleus was stained with DAPI (blue color). Quantitative analysis of Oil Red O staining (**h**) and BODIPY intensity (**i**) by Image J. n = 6. Quantitative analysis of intracardiac TG levels (**j**). n = 6. **k–m**, Heat map (left) and quantitative analysis (right) of lipid-targeted metabolomic of cardiac tissues from *db/db* TGR5^*fl/fl*^ and *db/db* TGR5^*ΔCM*^ mice at 20 weeks (**k**). n = 6. Red indicates upregulation and blue indicates downregulation. The columns and rows represent experimental heart samples and lipid species, respectively. Quantitative analysis of FFA levels in cardiac tissues from TGR5^*fl/fl*^ and TGR5^*ΔCM*^ mice in *db/m* or *db/db* background at 20 weeks (**l**). n = 6. FFA species in *db/db* TGR5^*fl/fl*^ and *db/db* TGR5^*ΔCM*^ mice (**m**). n = 6. Data are presented as mean ± SEM. Statistical significance was evaluated by two-tailed unpaired Student’s *t*-test (**h,i,k,m**) or two-way ANOVA followed by Tukey’s post-hoc test (**b,c,e,f,j,l**). Source data

To investigate alterations in the bile acid metabolism in DbCM, a mouse model of HFD combined with low-dose STZ (hereafter HFD/STZ) was utilized for 24 weeks (Fig. 1a and Supplementary Tables 1 and 2). Reduced levels of DCA were observed in the plasma and heart tissues of mice with diabetic myocardial injury (Fig. 1b,c). To explore the role of the DCA receptor TGR5 in DbCM, cardiac-specific TGR5-knockout (αMHC-*Gpbar1*^fl/fl^; TGR5^*ΔCM*^) mice (Supplementary Fig. 1a,b) were used in a diabetes model induced by HFD/STZ, representing late-stage type 2 diabetes. No significant differences in body weight, glucose tolerance, serum triglycerides (TGs) and fasting insulin levels were observed between TGR5^fl/fl^ and TGR5^*ΔCM*^ mice under healthy or diabetic conditions (Extended Data Fig. 1a–d). Ultrasound echocardiography revealed cardiac systolic and diastolic dysfunction in HFD/STZ-induced diabetic male and female mice, with TGR5^*ΔCM*^ mice showing decreased global longitudinal strain (GLS), ejection fraction (EF) and fraction shortening (FS), as well as an increased E/E′ ratio compared to TGR5^fl/fl^ mice (Fig. 1d–h and Extended Data Fig. 2a–e). In a genetic model of type 2 diabetes, we crossed *Gpbar1*^fl/fl^*;*αMHC-*cre* mice with *db/+* mice to obtain *db/db;*αMHC-*Gpbar1*^fl/fl^ (*db/db* TGR5^*ΔCM*^; Extended Data Fig. 3a). Consistently, echocardiography demonstrated significantly decreased cardiac function in *db/db* TGR5^*ΔCM*^ mice compared to *db/db* TGR5^fl/fl^ mice (Extended Data Fig. 3b–f). The echocardiographic measurements are summarized in Supplementary Tables 3–5. To investigate how TGR5 mediates the cardioprotective effects in diabetes, the morphology and molecular biology of the heart tissues were examined. Consistent with previous reports, hearts of mice with DbCM exhibited increased features of myocardial hypertrophy and fibrosis, including enlarged myocyte area, elevated protein levels of atrial natriuretic peptide (ANP), brain natriuretic peptide (BNP) and myosin heavy chain 7 (β-MHC), and enhanced accumulation of collagen fibres (Fig. 1i–m and Extended Data Fig. 1e). Furthermore, wheat germ agglutinin (WGA) staining, and immunoblotting of ANP, BNP and β-MHC indicated significant cardiomyocyte hypertrophy (Fig. 1i,j,l,m and Extended Data Fig. 2f,g), while Masson’s trichrome staining and collagen I content of the heart revealed aggravated myocardial fibrosis (Fig. 1i,k and Extended Data Figs. 1e and 2f,h) in TGR5^*ΔCM*^ mice compared to TGR5^fl/fl^ mice. These findings suggest that TGR5 deletion exacerbates DbCM induced by HFD/STZ or a *db/db* background.

### TGR5 deletion promotes myocardial lipid accumulation

**Fig. 2:**
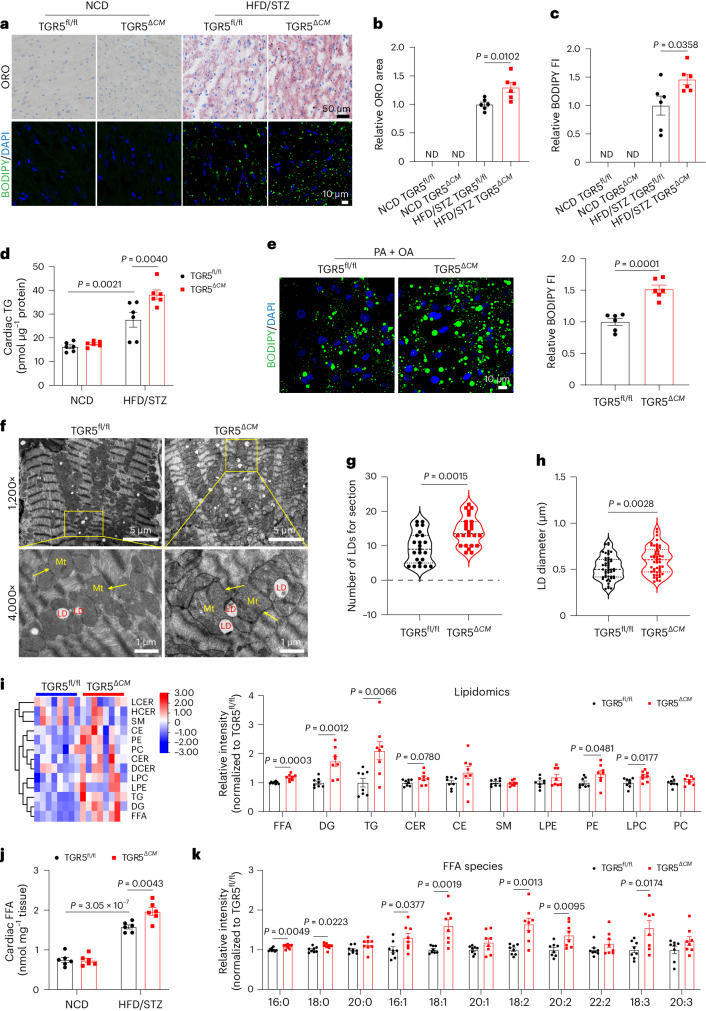
TGR5 deletion promotes myocardial lipid accumulation in mice with HFD/STZ-induced DbCM. **a**–**d**, Representative Oil Red O (ORO) and BODIPY 493/503 staining (green) images of cardiac tissues from TGR5^fl/fl^ and TGR5^*ΔCM*^ mice challenged with NCD or HFD/STZ for 24 weeks (**a**). Scale bars, 50 μm for Oil Red O staining; 10 μm for BODIPY 493/503 staining. The nucleus was stained with DAPI (blue). Quantification of Oil Red O staining (**b**) and BODIPY intensity (**c**) by ImageJ. *n* = 6. Quantification of intracardiac TG levels (**d**). *n* = 6. **e**, Representative BODIPY 493/503 staining images (left) and quantitative analysis (right) of primary NMCMs from TGR5^fl/fl^ and TGR5^*ΔCM*^ mice treated with PA (400 μmol l^−1^) + OA (200 μmol l^−1^) for 24 h. Scale bar, 10 μm. The nucleus was stained with DAPI. *n* = 6. **f**–**h**, Representative photomicrographs of the myocardium of TGR5^fl/fl^ and TGR5^*ΔCM*^ mice challenged with HFD/STZ for 24 weeks by TEM (**f**). Mt, mitochondria; LD, lipid droplet. Scale bars, 5 μm and 1 μm. Violin plot of lipid droplet (LD) number (**g**) and diameter (**h**) obtained using TEM analysis. *n* = 6. **i**–**k**, Heat map (left) and quantitative analysis (right) of lipid-targeted metabolomics of cardiac tissues from TGR5^fl/fl^ and TGR5^*ΔCM*^ mice challenged with HFD/STZ for 24 weeks (**i**). *n* = 8. Red indicates upregulation, and blue indicates downregulation. The columns and rows represent experimental heart samples and lipid species, respectively. Rows indicate ceramide (CER), cholesterol ester (CE), diglyceride (DG), lysophosphatidylcholine (LPC), lysophosphatidylethanolamine (LPE), phosphatidylcholine (PC), phosphatidylethanolamine (PE), sphingomyelin (SM) and TG. **j**, Quantification of FFA levels in cardiac tissues from TGR5^fl/fl^ and TGR5^*ΔCM*^ mice challenged with NCD or HFD/STZ. *n* = 6. **k**, FFA species in TGR5^fl/fl^ and TGR5^*ΔCM*^ mice challenged with HFD/STZ. *n* = 8. Data are presented as the mean ± s.e.m. Statistical significance was evaluated by two-tailed unpaired Student’s *t*-test (**b**, **c**, **e**, **h**, **i** and **k**), two-tailed non-parametric Mann–Whitney test (**g**) or two-way ANOVA followed by Tukey’s post hoc test (**d** and **j**). ND, not detected. Source data

To gain further insights into the underlying mechanisms of cardiac dysfunction phenotypes, we assessed the levels of lipid metabolism. Notably, intramyocardial lipid accumulation, as indicated by Oil Red O staining, BODIPY 493/503 staining (a lipid droplet probe) and TG levels, was increased in TGR5^*ΔCM*^ mice compared to TGR5^fl/fl^ mice subjected to HFD/STZ (Fig. 2a–d and Extended Data Fig. 2f,i,j) or mice in a genetic model of type 2 diabetes (Extended Data Fig. 3g–j). Consistent with these findings, we observed a significant increase of lipid droplets in TGR5^*ΔCM*^ primary neonatal mouse cardiomyocytes (NMCMs) treated with palmitic acid (PA) and oleic acid (OA) treatment compared to TGR5^fl/fl^ cardiomyocytes (Fig. 2e). Transmission electron microscopy (TEM) further revealed an increased number and size of lipid droplets in TGR5^*ΔCM*^ mice (Fig. 2f–h). Moreover, lipidomic analysis of myocardial tissue in TGR5^*ΔCM*^ mice under diabetic conditions showed distinct lipidomic profiles compared to TGR5^fl/fl^ mice. FFA, diacylglycerol (DG) and TG levels were significantly elevated in TGR5^*ΔCM*^ mice (Fig. 2i and Extended Data Fig. 3k). Specifically, FFA levels were markedly increased in TGR5^*ΔCM*^ mice (Fig. 2j and Extended Data Fig. 3l), with a significant increase in long-chain unsaturated fatty acids (Fig. 2k and Extended Data Fig. 3m). These findings suggest that TGR5 deficiency promotes myocardial lipid accumulation.

### TGR5 activation prevents cardiac dysfunction and cardiolipotoxicity

**Fig. 3:**
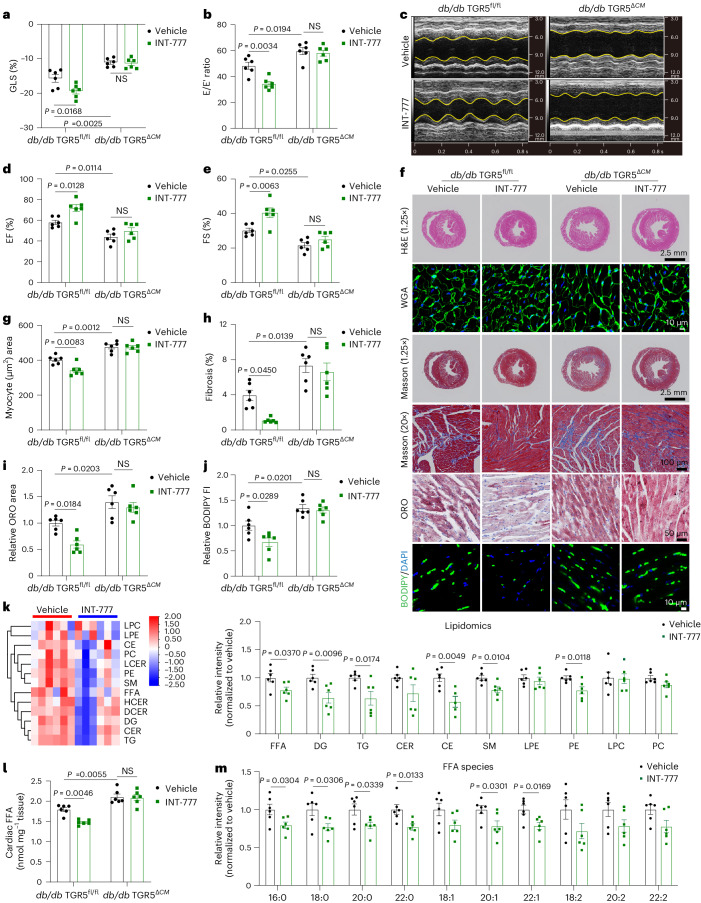
TGR5-specific agonist INT-777 prevents cardiac dysfunction and cardiolipotoxicity in *db/db* mice. **a**–**j**, *db/db* TGR5^fl/fl^ and *db/db* TGR5^*ΔCM*^ mice aged 20 weeks were treated with INT-777 (30 mg per kilogram body weight) for 12 weeks. Left ventricular GLS was calculated by Vevo software (**a**). *n* = 6. Ratio of flow Doppler E wave amplitude to tissue Doppler E′ wave amplitude (E/E′) (**b**). *n* = 6. Representative left ventricular M-mode echocardiographic images (**c**). Quantification of left ventricular EF (**d**) and FS (**e**). *n* = 6. Representative images of H&E, WGA, Masson’s trichrome, Oil Red O and BODIPY 493/503 (green) stainings of cardiac tissues from *db/db* TGR5^fl/fl^ and *db/db* TGR5^*ΔCM*^ mice with INT-777 or vehicle treatment for 12 weeks (**f**). Scale bars, 2.5 mm for H&E; 10 μm for WGA; 2.5 mm and 100 μm for Masson’s trichrome staining; 50 μm for Oil Red O staining; 10 μm for BODIPY 493/503 staining. The nucleus was stained with DAPI (blue). Quantification of myocyte area in WGA staining (**g**). *n* = 6. Quantification of cardiac fibrosis area in Masson’s trichrome staining (**h**). *n* = 6. Quantitative analysis of Oil Red O staining (**i**). *n* = 6. BODIPY fluorescence intensity (FI) by ImageJ (**j**). *n* = 6. **k**–**m**, Heat map (left) and quantitative analysis (right) of lipid-targeted metabolomics of primary NMCMs treated with PA + OA for 24 h in the presence or absence of INT-777 (**k**). *n* = 6. Red indicates upregulation, and blue indicates downregulation. The columns and rows represent experimental samples and lipid species, respectively. Quantitative analysis of FFA levels in cardiac tissues from *db/db* TGR5^fl/fl^ and *db/db* TGR5^*ΔCM*^ mice with INT-777 or vehicle treatment for 12 weeks (**l**). *n* = 6. FFA species in primary NMCMs treated with PA + OA for 24 h in the presence or absence of INT-777 (**m**). *n* = 6. Data are presented as the mean ± s.e.m. Statistical significance was evaluated by two-way ANOVA followed by Tukey’s post hoc test (**a**, **b**, **d**, **e**, **g**–**j** and **l**) or two-tailed unpaired Student’s *t*-test (**k** and **m**). NS, not significant. Source data

**Extended Data Fig. 4:**
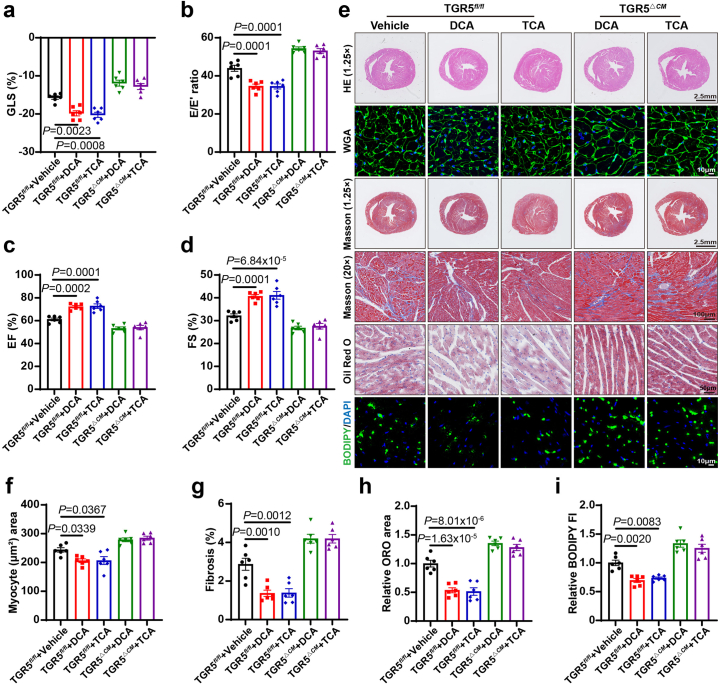
DCA and TCA prevent against cardiac dysfunction and cardiolipotoxicity in mice with HFD/STZ-induced diabetic cardiomyopathy. **a–d**, TGR5^*fl/fl*^ and TGR5^*ΔCM*^ mice challenged with HFD/STZ were treated with DCA or TCA for 12 weeks. Left ventricular GLS was calculated by Vevo Software (**a**). n = 6. Ratio of flow Doppler E wave amplitude to tissue Doppler E′ wave amplitude (E/E’) (**b**). n = 6. Quantification of left ventricular EF (**c**) and FS (**d**). n = 6. **e**, Representative images of H&E, WGA, Masson’s trichrome, Oil Red O and BODIPY 493/503 staining (green color) images of cardiac tissues from TGR5^*fl/fl*^ and TGR5^*ΔCM*^ mice with DCA, TCA or vehicle treatment for 12 weeks. Scale bar, 2.5 mm for H&E; 10 μm for WGA; 2.5 mm and 100 μm for Masson’s trichrome staining; 50 μm for Oil Red O staining; 10 μm for BODIPY 493/503 staining. The nucleus was stained with DAPI (blue color). **f**, Quantification of myocyte area in WGA staining. n = 6. **g**, Quantification of cardiac fibrosis area in Masson’s trichrome staining. n = 6. **h**, Quantitative analysis of Oil Red O staining. n = 6. **i**, BODIPY intensity by Image J. n = 6. Data are presented as mean ± SEM. Statistical significance was evaluated by one-way ANOVA followed by Tukey’s post-hoc test. Source data

Additionally, to evaluate the impact of TGR5 on preventing cardiolipotoxicity, *db/db* mice were treated with the TGR5 agonist INT-777 via gavage for 12 weeks. As anticipated, INT-777 administration improved cardiac systolic and diastolic function in *db/db* mice compared to the vehicle-treated group, but this effect was not observed in *db/db* TGR5^*ΔCM*^ mice (Fig. 3a–e and Supplementary Table 6). Furthermore, INT-777 significantly inhibited cardiomyocyte hypertrophy and fibrosis in the hearts of *db/db* mice, as evidenced by WGA and Masson’s trichrome staining (Fig. 3f–h). INT-777-treated *db/db* mice exhibited reduced lipid accumulation, as indicated by Oil Red O staining and BODIPY 493/503 staining, whereas INT-777 treatment had no significant influence on lipid metabolism in *db/db* TGR5^*ΔCM*^ mice (Fig. 3f,i,j). Consistent with INT-777 treatment, HFD/STZ-induced mice treated with bile acid DCA or TCA improved cardiac function and lipid accumulation in a TGR5-dependent manner (Extended Data Fig. 4). These findings suggest that the activation of cardiac TGR5 may inhibit myocardial lipid accumulation. The beneficial effect of INT-777 on lipid accumulation in primary NMCMs treated with PA + OA was further confirmed through lipidomic analysis. FFA, DG and TG levels were significantly reduced in INT-777-treated primary NMCMs compared to the vehicle-treated group (Fig. 3k). Quantitative analysis also revealed decreased FFA levels in INT-777-treated mice (Fig. 3l). Furthermore, FFA species analysis showed a significant decrease in long-chain fatty acids (Fig. 3m). These results indicate that TGR5 could be a potential therapeutic target, and TGR5 activation can prevent cardiolipotoxicity and cardiac dysfunction.

### TGR5 inhibits fatty acid uptake and PM localization of CD36

**Fig. 4:**
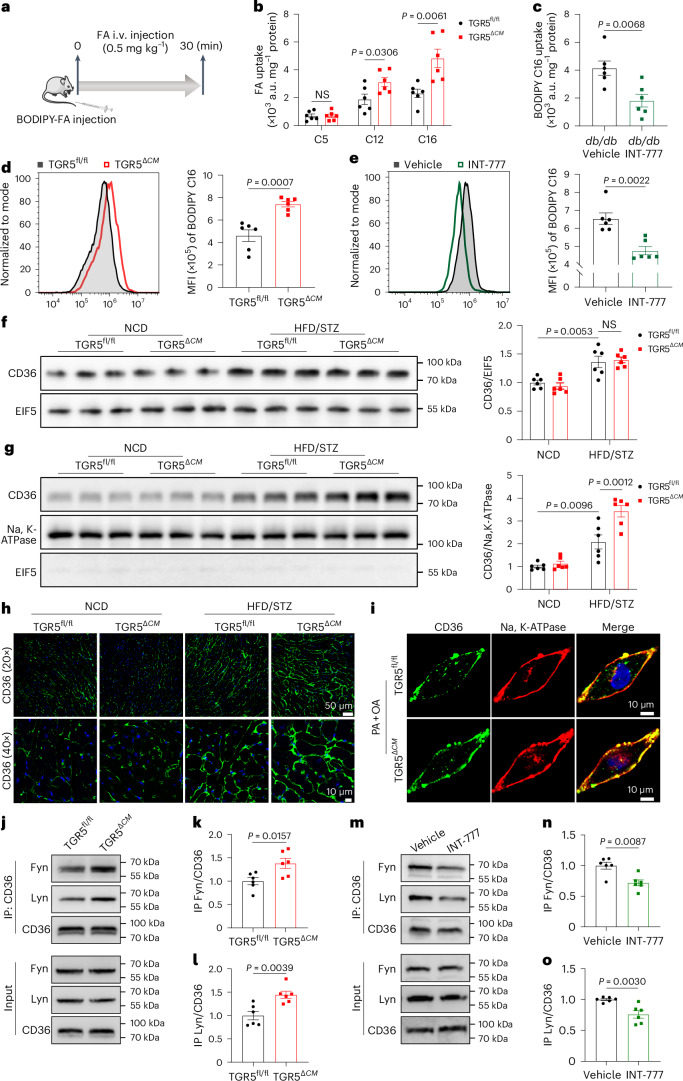
TGR5 reduces long-chain fatty acid uptake and localization of CD36 on the PM of cardiomyocytes. **a**, Schematic showing the procedure of myocardial tissue uptake of intravenously (i.v) administered BODIPY fluorescent-conjugated fatty acids. Created with BioRender.com with modifications. **b**, Comparisons of short-chain fatty acid (BODIPY C5) and long-chain fatty acid (BODIPY C12 and C16) uptake in TGR5^fl/fl^ and TGR5^*ΔCM*^ mice challenged with HFD/STZ for 24 weeks. *n* = 6. **c**, Comparisons of BODIPY C16 uptake in *db/db* mice treated with INT-777 or vehicle for 12 weeks. *n* = 6. **d**, Representative flow diagram (left) and comparisons (right) of fatty acid (BODIPY C16) uptake by primary NMCMs from TGR5^fl/fl^ and TGR5^*ΔCM*^ mice treated with PA + OA for 24 h. *n* = 6. **e**, Representative flow diagram (left) and comparisons (right) of BODIPY C16 uptake by NMCMs treated with PA + OA for 24 h in the presence or absence of INT-777. *n* = 6. **f**, Representative western blot images (left) and comparisons (right) of the total protein level of CD36 in cardiac tissues. *n* = 6. **g**, Representative western blot images (left) and comparisons (right) of CD36 plasma membrane protein isolated from cardiac tissues. *n* = 6. **h**,**i**, Representative confocal images of endogenous CD36 (green) and Na^+^/K^+^-ATPase (red) detected by the anti-CD36 and anti-Na^+^/K^+^-ATPase antibodies conjugated with Alexa Fluor 488 and Alexa Fluor 568 in vivo (**h**) and in vitro (**i**), respectively. Scale bars, 50 μm and 10 μm for cardiac tissue; 10 μm for cardiomyocytes. *n* = 6. **j**–**l**, Representative co-immunoprecipitation (IP) images (**j**) and comparisons of Fyn (**k**), Lyn (**l**) and CD36 in PA + OA-treated primary NMCMs from TGR5^fl/fl^ and TGR5^*ΔCM*^ mice for 24 h. *n* = 6. **m**–**o**, Representative co-immunoprecipitation images (**m**) and comparisons of Fyn (**n**), Lyn (**o**) and CD36 in primary NMCMs treated with PA + OA for 24 h in the presence or absence of INT-777. *n* = 6. Data are presented as the mean ± s.e.m. Statistical significance was evaluated by two-tailed unpaired Student’s *t*-test (**b**–**d**, **k**, **l** and **o**), two-tailed non-parametric Mann–Whitney test (**e** and **n**) or two-way ANOVA followed by Tukey’s post hoc test (**f** and **g**). a.u., arbitrary units; MFI, mean fluorescence intensity. Source data

**Extended Data Fig. 5:**
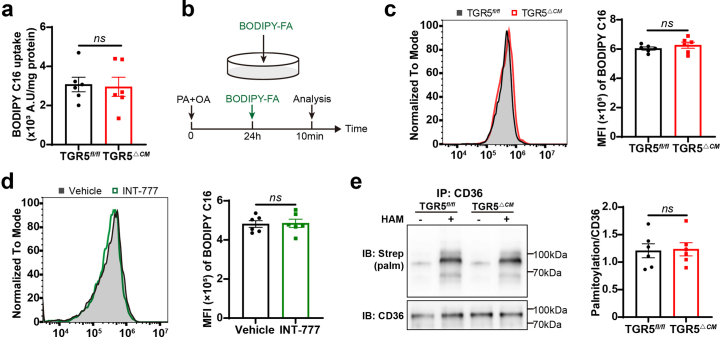
TGR5 does not affect fatty acid uptake under normal physiological conditions. **a**, Comparisons of BODIPY C-16 uptake in TGR5^*fl/fl*^ and TGR5^*ΔCM*^ mice under physiological conditions. n = 6. **b**, A schematic diagram showing the procedure of cardiomyocyte uptake of administered BODIPY fluorescent-conjugated fatty acids. **c**, Representative flow diagram (left) and comparisons (right) of BODIPY C-16 uptake by primary NMCMs from TGR5^*fl/fl*^ and TGR5^*ΔCM*^ mice. n = 6. **d**, Representative flow diagram (left) and comparisons (right) of BODIPY C-16 uptake by primary NMCMs in the presence or absence of INT-777. n = 6. **e**, Representative western blot images (left) and comparisons (right) of CD36 palmitoylation in cardiac tissues. CD36 palmitoylation was determined in TGR5^*fl/fl*^ and TGR5^*ΔCM*^ mice under physiological conditions. n = 6. Data are presented as mean ± SEM. Statistical significance was evaluated by two-tailed unpaired Student’s *t*-test. Source data

**Extended Data Fig. 6:**
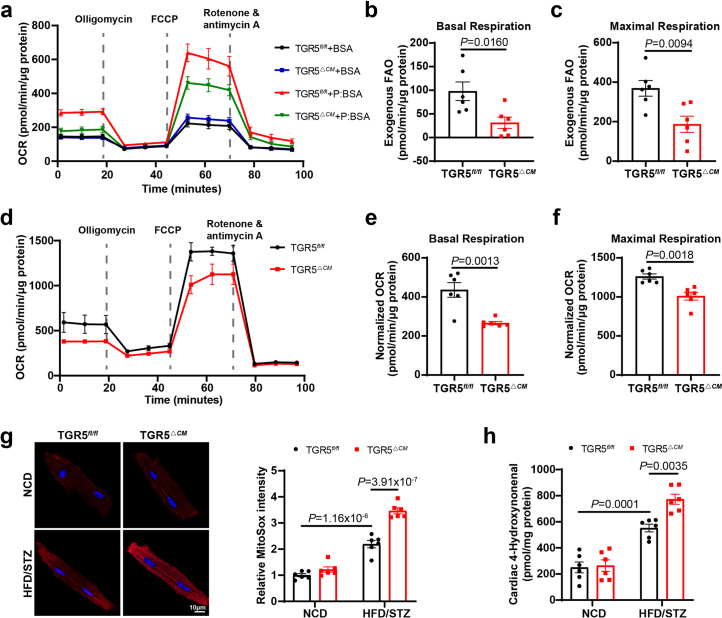
TGR5 deletion aggravates cardiac mitochondrial dysfunction in mice of HFD/STZ-induced diabetic cardiomyopathy. **a–c**, Oxygen consumption rate (OCR) was used to evaluate fatty acid oxidation (FAO) activity due to the utilization of exogenous palmitate-conjugated BSA (P:BSA) or BSA in primary NMCMs from TGR5^*fl/fl*^ and TGR5^*ΔCM*^ mice treated with PA + OA for 24 h (**a**). Quantitative analysis of basal respiration (**b**) and maximal respiration (**c**). n = 6. FAO-dependent mitochondrial respiration was measured as oxygen consumption rate differences (∆OCR) with or without palmitate. ∆OCR demonstrated that the preponderance of respiration resulted from FAO. **d–f**, Mitochondrial oxidative respiration in primary NMCMs from TGR5^*fl/fl*^ and TGR5^*ΔCM*^ mice treated with PA + OA for 24 h as assessed by measuring the cellular OCR. Quantitative analysis of basal respiration (**e**) and maximal respiration (**f**). n = 6. Basal respiration is derived from (last rate measurement before first injection) - (non-mitochondrial respiration) and maximal respiration is derived from (maximum rate measurement after FCCP injection) - (non-mitochondrial respiration). **g**, Mitochondrial ROS levels were detected in isolated cardiomyocytes from TGR5^*fl/fl*^ and TGR5^*ΔCM*^ mice challenged with NCD or HFD/STZ using MitoSox probe (red). The nucleus was stained with Hoechst (blue color). **h**, Quantification of cardiac 4-Hydroxynonenal (4-HNE) levels in cardiac tissues from TGR5^*fl/fl*^ and TGR5^*ΔCM*^ mice challenged with NCD or HFD/STZ. n = 6. Data are presented as mean ± SEM. Statistical significance was evaluated by two-tailed unpaired Student’s *t*-test (**b,c,e,f**) or two-way ANOVA followed by Tukey’s post-hoc test (**g,h**). Source data

Considering the unique nature of cardiac lipid metabolism, fatty acid uptake and FAO were evaluated. We investigated the impact of TGR5 on fatty acid trafficking into the heart by measuring myocardial tissue uptake of intravenously administered BODIPY fluorescent-conjugated fatty acids in TGR5^*ΔCM*^ and TGR5^fl/fl^ mice (Fig. 4a). Compared to TGR5^fl/fl^ mice, although there was no difference under normal physiological conditions (Extended Data Fig. 5a–d), diabetic TGR5^*ΔCM*^ mice exhibited increased fatty acid uptake by myocardial tissue, particularly for long-chain fatty acids (Fig. 4b). Treatment of *db/db* mice with the TGR5 agonist INT-777 resulted in decreased uptake of long-chain fatty acids (Fig. 4c). In vitro experiments with isolated TGR5^fl/fl^ and TGR5^*ΔCM*^ cardiomyocytes and primary NMCMs treated with INT-777 confirmed the inhibitory effect of TGR5 on fatty acid uptake (Fig. 4d,e). To evaluate the effect of TGR5 on FAO, we performed the Seahorse palmitate oxidation stress test. By performing oxygen consumption rate (OCR) assays in the presence of exogenous palmitate, TGR5-deficient cardiomyocytes were shown to reduce utilization of exogenous fatty acids to drive basal and maximum respiration (Extended Data Fig. 6a–c). Additionally, mitochondrial function, assessed through Seahorse analysis, was significantly reduced in primary NMCMs isolated from TGR5^*ΔCM*^ mice compared to TGR5^fl/fl^ mice (Extended Data Fig. 6d–f). Moreover, TGR5^*ΔCM*^ mice exhibited increased MitoSox and 4-hydroxynonenal (4-HNE) levels in myocardial tissue (Extended Data Fig. 6g,h), indicating impaired mitochondrial function. These results suggest that TGR5 inhibits fatty acid uptake and helps maintain FAO and mitochondrial function in cardiomyocytes.

**Extended Data Fig. 7:**
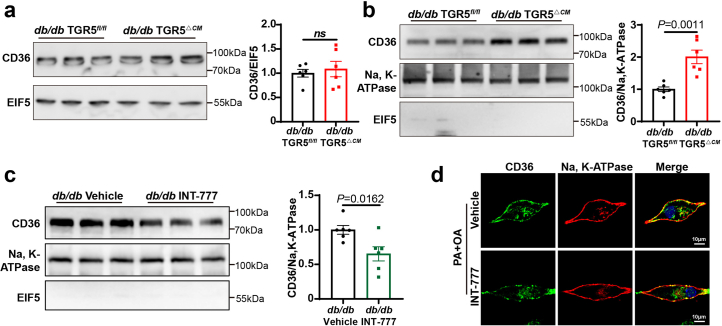
TGR5 inhibits the localization of CD36 on the PM of cardiomyocytes. **a**, Representative western blot images (left) and comparisons (right) of the total protein level of CD36 in cardiac tissues of *db/db* TGR5^*fl/fl*^ and *db/db* TGR5^*ΔCM*^ mice at 20 weeks. n = 6. **b,c**, Representative western blot images (left) and comparisons (right) of CD36 plasma membrane protein isolated from cardiac tissues of *db/db* TGR5^*fl/fl*^ and *db/db* TGR5^*ΔCM*^ mice at 20 weeks (**b**), and *db/db* mice treated with INT-777 for 12 weeks (**c**). n = 6. **d**, Representative confocal images of endogenous CD36 (green color) and Na^+^/K^+^-ATPase (red color) detected by the anti-CD36 and anti-Na^+^/K^+^-ATPase antibodies conjugated with Alexa Fluor 488 and Alexa Fluor 568, respectively. Scale bar, 10 μm. n = 6. Data are presented as mean ± SEM. Statistical significance was evaluated by two-tailed unpaired Student’s *t*-test. Source data

CD36, the primary mediator of long-chain fatty acid uptake in the heart, relies on its expression and localization on the plasma membrane (PM) for proper function^6,23^. Therefore, we examined the total protein and membrane protein expression of CD36 in the hearts of HFD/STZ-induced and *db/db* mice. The total protein level of CD36 was not significantly different between TGR5^*ΔCM*^ mice and TGR5^fl/fl^ mice (Fig. 4f). However, TGR5^*ΔCM*^ mice exhibited elevated levels of PM-localized CD36 compared to TGR5^fl/fl^ mice (Fig. 4g,h). Similar observations were made in *db/db* TGR5^*ΔCM*^ mice compared to *db/db* TGR5^fl/fl^ mice (Extended Data Fig. 7a,b). Treatment of *db/db* mice with the TGR5 agonist INT-777 for 12 weeks, led to a significant reduction in the PM localization of CD36 in the heart (Extended Data Fig. 7c). In primary NMCMs from TGR5^*ΔCM*^ mice and NMCMs treated with INT-777, induction of PA + OA resulted in increased PM localization of CD36 in TGR5^*ΔCM*^ cells and decreased localization in INT-777-treated cells, confirming the in vivo findings (Fig. 4i and Extended Data Fig. 7d).

In addition, CD36 recruits the Src family kinases Fyn and Lyn to assemble signalling protein complexes to activate downstream signalling pathways, which functions independently of fatty acid transport^24,25^. We investigated whether TGR5 is involved in modulating the formation of the CD36–Fyn–Lyn complex. TGR5 deletion significantly increased the expression of Fyn and Lyn detected in CD36 immunoprecipitation in primary NMCMs treated with PA + OA (Fig. 4j–l). Conversely, INT-777 treatment reversed these effects (Fig. 4m–o). These data indicate that TGR5 inhibits the assembly of the CD36–Fyn–Lyn complex. Together, these findings provide partial explanations for the upregulated fatty acid uptake observed due to increased CD36 localization on the PM in the hearts of TGR5^*ΔCM*^ mice.

### TGR5–DHHC4 signalling regulates CD36 palmitoylation

**Fig. 5:**
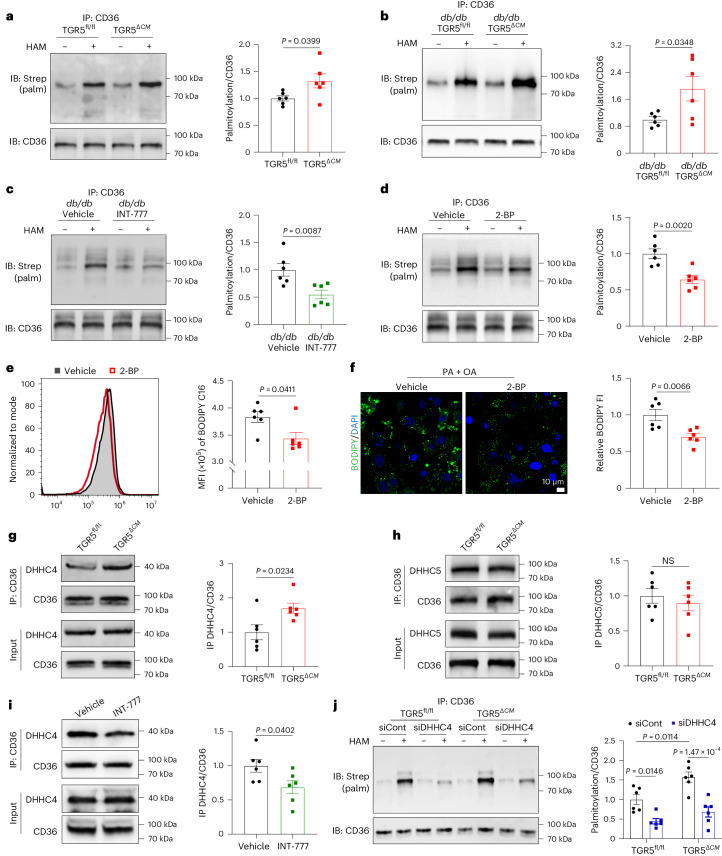
TGR5 inhibits CD36 palmitoylation via DHHC4. **a**–**c**, Representative western blot images (left) and comparisons (right) of CD36 palmitoylation in cardiac tissues. CD36 palmitoylation was determined in TGR5^fl/fl^ and TGR5^*ΔCM*^ mice challenged with HFD/STZ for 24 weeks (**a**), *db/db* TGR5^fl/fl^ and *db/db* TGR5^*ΔCM*^ mice at 20 weeks (**b**), or *db/db* mice treated with INT-777 or vehicle for 12 weeks (**c**) by ABE assay. *n* = 6. **d**, Representative western blot images (left) and comparisons (right) of CD36 palmitoylation in cardiomyocytes. Primary NMCMs were co-incubated with PA + OA and 2-BP (100 μmol l^−1^) for 24 h. CD36 palmitoylation was determined by ABE assay. *n* = 6. **e**, Representative flow diagram (left) and comparisons (right) of BODIPY C16 uptake by primary NMCMs co-incubated with PA + OA and 2-BP for 24 h. *n* = 6. **f**, Representative BODIPY 493/503 staining images (left) and quantitative analysis (right) of primary NMCMs treated with PA + OA for 24 h in the presence or absence of 2-BP. Scale bar, 10 μm. *n* = 6. **g**,**h**, Representative co-immunoprecipitation images (left) and comparisons (right) of CD36 and DHHC4 (**g**) or DHHC5 (**h**) in PA + OA-treated primary NMCMs from TGR5^fl/fl^ and TGR5^*ΔCM*^ mice for 24 h. *n* = 6. **i**, Representative co-immunoprecipitation images (left) and comparisons (right) of CD36 and DHHC4 in primary NMCMs treated with PA + OA for 24 h in the presence or absence of INT-777. *n* = 6. **j**, Representative western blot images (left) and comparisons (right) of palmitoylated CD36 in cardiomyocytes. PA + OA were incubated for 24 h in primary NMCMs from TGR5^fl/fl^ and TGR5^*ΔCM*^ mice transfected with DHHC4 siRNA (siDHHC4) or scramble siRNA (siCont). CD36 palmitoylation was determined by ABE assay. *n* = 6. Data are presented as the mean ± s.e.m. Statistical significance was evaluated by two-tailed unpaired Student’s *t*-test (**a**, **b**, **d** and **f**–**i**), two-tailed non-parametric Mann–Whitney test (**c** and **e**) or two-way ANOVA followed by Tukey’s post hoc test (**j**). Source data

**Extended Data Fig. 8:**
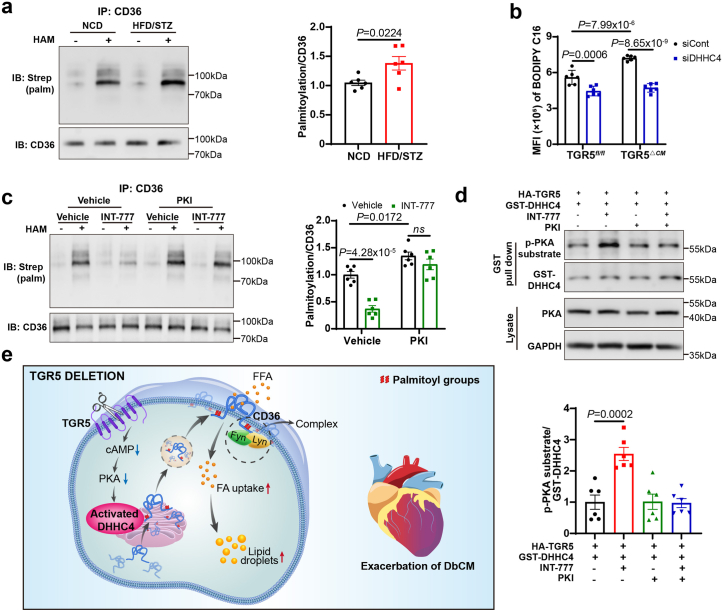
The inhibitory effect of TGR5 on DHHC4 is cAMP-PKA-dependent. **a**, Representative western blot images (left) and comparisons (right) of CD36 palmitoylation in cardiac tissues. CD36 palmitoylation was determined in mice treated with NCD and HFD/STZ for 24 weeks. n = 6. **b**, Flow comparisons of BODIPY C-16 uptake in primary NMCMs from TGR5^*fl/fl*^ and TGR5^*ΔCM*^ mice transfected with siDHHC4 or siCont and treated with PA + OA for 24 h. n = 6. **c**, Representative western blot images of palmitoylated CD36 in cardiomyocytes. PA + OA were incubated for 24 h in primary NMCMs in the presence or absence of INT-777 and PKA inhibitor PKI. CD36 palmitoylation was determined by ABE assay. n = 6. **d**, HEK293A cells were transfected with the indicated vectors and treated with 1μmol/L PKI for 1 h before stimulation with INT-777 for 6 h. DHHC4 was then enriched by GST pull-down. Phosphorylation of DHHC4 was detected using p-PKA substrate antibody. n = 6. **e**, The graphical summary mediated by TGR5 in the regulation of DbCM. Briefly, TGR5 deficiency results in the suppression of cAMP/PKA signaling in diabetes. Inactivated PKA reduces the phosphorylation of substrate DHHC4 and activates DHHC4, resulting in increased CD36 palmitoylation. Palmitoacylated CD36 is localized to the plasma membrane, thus promoting FFA uptake and aggravating cardiolipotoxicity. Created with BioRender.com with modifications. Data are presented as mean ± SEM. Statistical significance was evaluated by two-tailed unpaired Student’s *t*-test (**a**), two-way ANOVA followed by Tukey’s post-hoc test (**b,c**), or one-way ANOVA followed by Tukey’s post-hoc test (**d**). Source data

Palmitoylation is a post-translational modification that plays a crucial role in regulating the subcellular distribution and function of proteins by enhancing their lipophilicity^26^. Given its association with PM localization of membrane proteins, we investigated the level of CD36 palmitoylation using an acyl-biotin exchange (ABE) assay on proteins extracted from the hearts of HFD/STZ-induced and *db/db* mice. Our results revealed increased levels of palmitoylated CD36 in the hearts of diabetic mice (Extended Data Fig. 8a). While TGR5 deletion did not affect CD36 palmitoylation under normal physiological conditions (Extended Data Fig. 5e), TGR5^*ΔCM*^ mice exhibited higher levels of CD36 palmitoylation than TGR5^fl/fl^ mice in HFD/STZ-induced diabetes (Fig. 5a). Similarly, we observed the results in *db/db* TGR5^*ΔCM*^ and *db/db* TGR5^fl/fl^ mice (Fig. 5b). Additionally, we investigated the impact of a TGR5 receptor agonist on CD36 palmitoylation. Treatment with INT-777 significantly decreased CD36 palmitoylation compared to vehicle-treated *db/db* mice (Fig. 5c). To examine the effect of CD36 palmitoylation on fatty acid uptake and lipid accumulation in cardiomyocytes, we used 2-bromopalmitate (2-BP), a known inhibitor of palmitoyl acyltransferase^27^, to block palmitoylation (Fig. 5d). Primary NMCMs were co-incubated with PA + OA and 2-BP or PA + OA alone, and fatty acid uptake and lipid accumulation were assessed using flow cytometry and BODIPY 493/503 staining, respectively. Our results demonstrated that inhibition of palmitoylation significantly reduced fatty acid uptake and lipid accumulation in cardiomyocytes (Fig. 5e,f). These findings indicate that TGR5 inhibits the palmitoylation of CD36.

Protein palmitoylation is catalysed by a group of zinc finger DHHC domain-containing palmitoyl acyltransferases (DHHCs), which add a palmitoyl group to the thiol group of cysteine residues^28^. CD36 palmitoylation, mediated by DHHC4 and DHHC5, is primarily responsible for its localization on the PM and subsequent fatty acid uptake^29^. To identify potential palmitoyl transferases involved in the TGR5 pathway, we performed co-immunoprecipitation assays to investigate the interactions between CD36 and DHHC4 or DHHC5. The results showed that TGR5 deletion enhanced the binding between CD36 and DHHC4 (Fig. 5g) but did not alter the binding between CD36 and DHHC5 (Fig. 5h). Treatment with INT-777 significantly inhibited the association between CD36 and DHHC4 expression (Fig. 5i). To further evaluate the correlation between TGR5 and DHHC4, we used siRNA for KD of DHHC4 in PA + OA-stimulated primary NMCMs. Our findings revealed that siDHHC4 significantly reduced CD36 palmitoylation compared to siCont, indicating that DHHC4 is involved in CD36 palmitoylation (Fig. 5j). Moreover, in TGR5-deficient cardiomyocytes, CD36 palmitoylation was significantly reduced when DHHC4 was silenced, suggesting that the effect of TGR5 on CD36 palmitoylation is dependent on DHHC4 (Fig. 5j). Analysis of fatty acid uptake also confirmed that the inhibitory effect of TGR5 on CD36 function is DHHC4 dependent (Extended Data Fig. 8b). As a G-protein-coupled receptor, TGR5 mediates its effects through the classic cAMP–protein kinase A (PKA) signalling pathway^10,30^. To determine whether the inhibition of DHHC4 by TGR5 is cAMP–PKA dependent, we treated primary NMCMs with INT-777 and the PKA inhibitor PKI. Our results showed that the inhibitory effect of TGR5 activation on CD36 palmitoylation was blocked by PKA inhibition (Extended Data Fig. 8c). To further confirm that DHHC4 is a phosphorylated substrate for endogenous PKA, INT-777 was used to activate endogenous PKA. INT-777 promoted the phosphorylation of DHHC4. The effect was abolished by the specific PKA inhibitor PKI (Extended Data Fig. 8d), indicating that endogenous PKA phosphorylated DHHC4 in vivo. These data suggest that TGR5–DHHC4 signalling mediates CD36 palmitoylation in cardiomyocytes.

### DHHC4 KD rescues cardiac dysfunction in TGR5-deficient mice

**Fig. 6:**
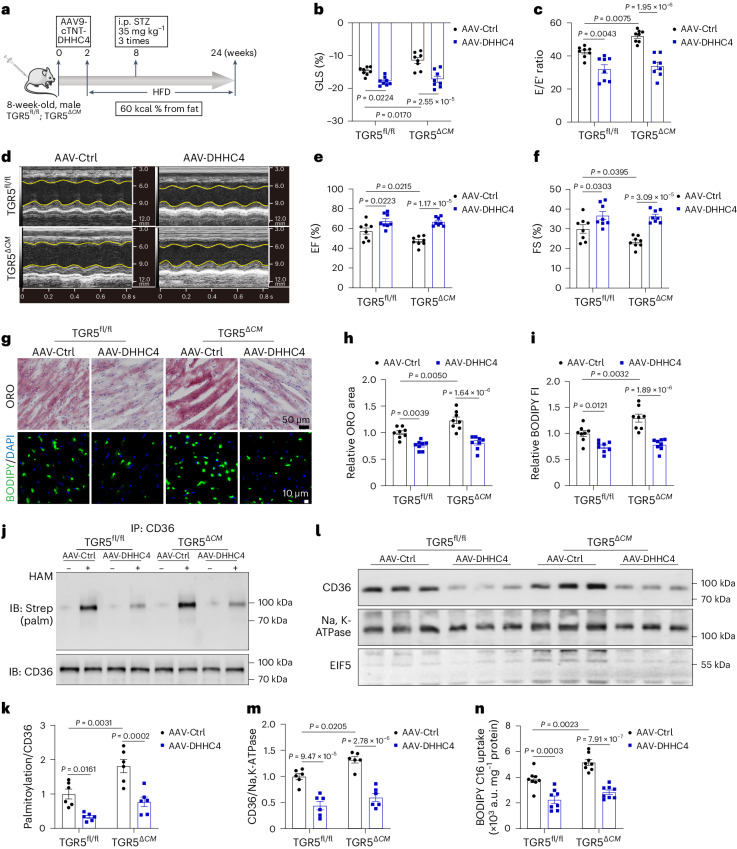
DHHC4 KD ameliorates the deleterious effect of TGR5 deficiency in mice with HFD/STZ-induced DbCM. **a**, Eight-week-old TGR5^fl/fl^ and TGR5^*ΔCM*^ mice were injected with adeno-associated virus of serotype 9 (AAV9) through a tail vein to knock down DHHC4 under the transcriptional control of cardiomyocyte-specific troponin T promoter (5 × 10^12^ plaque-forming units per mouse). At 2 weeks after AAV9 injection, the mice were fed with HFD for 24 weeks. At week 8 after HFD feeding, the mice were injected with STZ (35 mg per kg body weight) for 3 consecutive days to induce diabetes. Created with BioRender.com with modifications. **b**–**f**, Left ventricular GLS was calculated using Vevo software (**b**). *n* = 8. Ratio of flow Doppler E wave amplitude to tissue Doppler E′ wave amplitude (E/E′) (**c**). *n* = 8. Representative left ventricular M-mode echocardiographic images (**d**). Quantification of left ventricular EF (**e**). *n* = 8. Quantification of left ventricular FS (**f**). *n* = 8. **g**–**i**, Representative Oil Red O and BODIPY 493/503 staining (green) images of cardiac tissues from HFD/STZ TGR5^fl/fl^ and TGR5^*ΔCM*^ mice with AAV-DHHC4 or AAV-Ctrl treatment (**g**). Scale bars, 50 μm for Oil Red O staining; 10 μm for BODIPY 493/503 staining. The nucleus was stained with DAPI (blue). Quantitative analysis of Oil Red O staining (**h**) and BODIPY intensity (**i**) by ImageJ. *n* = 8. **j**,**k**, Representative western blot images (**j**) and comparisons (**k**) of CD36 palmitoylation in HFD/STZ TGR5^fl/fl^ and TGR5^*ΔCM*^ mice with AAV-DHHC4 or AAV-Ctrl treatment. *n* = 6. **l**,**m**, Representative western blot images (**l**) and comparisons (**m**) of CD36 plasma membrane protein isolated from cardiac tissues of HFD/STZ TGR5^fl/fl^ and TGR5^*ΔCM*^ mice with AAV-DHHC4 or AAV-Ctrl treatment. *n* = 6. **n**, Comparisons of BODIPY C16 uptake in HFD/STZ TGR5^fl/fl^ and TGR5^*ΔCM*^ mice with AAV-DHHC4 or AAV-Ctrl treatment. *n* = 8. Data are presented as the mean ± s.e.m. Statistical significance was evaluated by two-way ANOVA followed by Tukey’s post hoc test. Source data

To further evaluate the role of DHHC4 in vivo, we generated cardiomyocyte-specific DHHC4-KD mice by administering AAV9-cTNT-DHHC4 via tail vein infusion in HFD/STZ-induced TGR5^fl/fl^ and TGR5^*ΔCM*^ mice (Fig. 6a and Supplementary Fig. 3). Ultrasound echocardiography revealed that DHHC4 KD effectively ameliorated HFD/STZ-induced cardiac dysfunction and reversed the deterioration of cardiac function observed in TGR5-deleted mice (Fig. 6b–f and Supplementary Table 7). Moreover, we observed a significant reduction in myocardial lipid accumulation in HFD/STZ-induced TGR5^fl/fl^ and TGR5^*ΔCM*^ mice after DHHC4 KD (Fig. 6g–i). At the molecular level, DHHC4 KD led to decreased palmitoylation and PM localization of CD36 in the heart (Fig. 6j–m). Functionally, DHHC4 KD resulted in a significant reduction in fatty acid uptake (Fig. 6n). These findings indicate that the impact of TGR5 deletion on increased lipid accumulation and cardiac dysfunction is dependent on DHHC4 activation.

### Association of DCA in participants with diabetes with myocardial injury

**Fig. 7:**
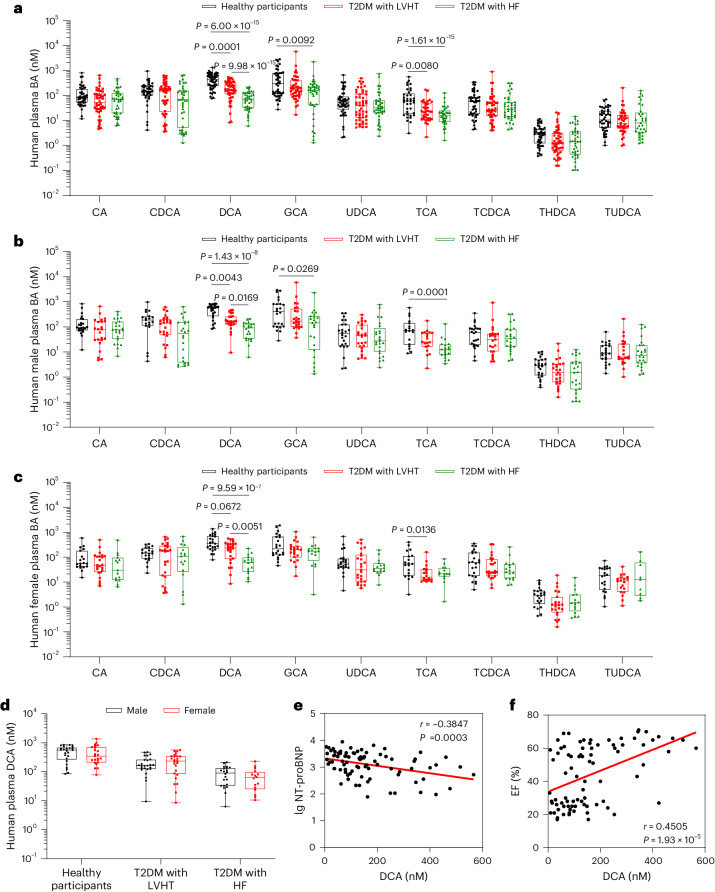
Decreased levels of TGR5-biased bile acids are associated with diabetic myocardial injury. **a**, Bile acid profiles in collected clinical plasma samples from healthy participants (*n* = 48), T2DM with LVHT (*n* = 50) and T2DM with HF (*n* = 42). **b**, Bile acid profiles in males from healthy participants (*n* = 25), T2DM with LVHT (*n* = 25) and T2DM with HF (*n* = 25). **c**, Bile acid profiles in females from healthy participants (*n* = 23), T2DM with LVHT (*n* = 25) and T2DM with HF (*n* = 17). **d**, Comparison of bile acid DCA levels in males (healthy participants for *n* = 25; T2DM with LVHT for *n* = 25; T2DM with HF for *n* = 25) and females (healthy participants for *n* = 23; T2DM with LVHT for *n* = 25; T2DM with HF for *n* = 17). **e**,**f**, The correlation analysis between plasma DCA levels and N-terminal pro-B-type natriuretic peptide (NT-proBNP) levels (**e**), and between plasma DCA levels and left ventricular EF (**f**) in participants with diabetic myocardial injury. *n* = 84. In the box plots, the midline represents the median, the box represents the IQR between the first and third quartiles, and whiskers represent the lowest or highest values within 1.5 times the IQR from the first or third quartiles. Statistical significance was evaluated by Kruskal–Wallis test (**a**–**c**) or two-tailed non-parametric Spearman correlation (**e** and **f**). Source data

To investigate the potential alterations in bile acid levels associated with diabetic myocardial injury, we conducted a comprehensive analysis of plasma bile acids in healthy participants, as well as participants with type 2 diabetes mellitus (T2DM) presenting with left ventricular hypertrophy (LVHT) or heart failure (HF) using bile acid-targeted metabolomics (Supplementary Table 9). Our analysis revealed significant changes in specific bile acid species, specifically DCA and TCA, which are known to exhibit bias towards the TGR5 receptor. Compared to healthy participants, participants with diabetic LVHT exhibited reduced levels of DCA, which were further decreased in participants with diabetic HF (Fig. 7a). Considering that women with diabetes take a steeper path to cardiomyopathy and HF^31,32^, we divided the bile acid profiles by sex. In both male and female participants, DCA levels were significantly reduced in participants with diabetic LVHT and diabetic HF (Fig. 7b,c), but there was no significant difference between the sexes (Fig. 7d). Furthermore, correlation analysis demonstrated a negative association between plasma DCA levels and N-terminal pro-B-type natriuretic peptide levels (Fig. 7e), while a positive correlation was observed between DCA levels and EF (Fig. 7f). Collectively, these findings highlight the involvement of the bile acid–TGR5 pathway in the development of DbCM and its potential as a diagnostic marker for the condition.

## Discussion

Cardiolipotoxicity, characterized by excessive lipid accumulation in the heart, is a major contributor to cardiac remodelling and dysfunction in individuals with diabetes^33^. Therefore, targeting cardiolipotoxicity has emerged as a promising therapeutic strategy for DbCM. In this study, we investigated the role of TGR5, a bile acid receptor, in regulating myocardial lipid uptake and its impact on DbCM progression and lipid metabolism. Our findings revealed a significant decrease in levels of TGR5-biased bile acid DCA in participants and mice with diabetic myocardial injury. TGR5 acts as a key regulator of myocardial lipid uptake by inhibiting the palmitoylation and PM localization of CD36, a primary fatty acid transporter. We utilized cardiomyocyte-specific TGR5-deficient mice to assess the effects of TGR5 on DbCM. Interestingly, TGR5 deletion resulted in increased fatty acid uptake and lipid accumulation in the heart, exacerbating cardiac hypertrophy, fibrosis and dysfunction in DbCM mouse models. Further investigation showed that TGR5 deletion enhanced CD36 palmitoylation through the action of the palmitoyl acyltransferase DHHC4, promoting the PM localization of CD36 in cardiomyocytes. Importantly, these detrimental effects of excessive lipid accumulation and cardiac remodelling were effectively reversed by administration of the TGR5 agonist INT-777 or bile acids DCA and TCA. Our findings highlight the crucial role of the TGR5–DHHC4 pathway in mediating fatty acid uptake via CD36, providing protection against cardiac lipotoxicity and the development of DbCM.

Bile acids and their receptors play vital roles in various metabolic and cardiovascular processes^34^. Interestingly, plasma bile acid levels, especially those biased towards TGR5, have been paradoxically elevated in individuals with diabetes^12,13^. In our study, we observed a significant decrease in DCA levels as participants with diabetes progressed to LVHT and HF, possibly due to alterations in the gut microbiota. DCA, an endogenous ligand for TGR5, has been previously shown to exert protective effects in the heart^20,35^. The reduction of DCA in participants with diabetic myocardial hypertrophy and HF indicates impaired TGR5 signalling. TGR5 activation plays a pivotal role in modulating calcium signalling, inflammatory responses and oxidative stress, thereby mitigating hyperglycaemia-induced damage to cardiomyocytes^36,37^. While the metabolic role of TGR5 has been extensively studied in other tissues, its involvement in cardiac metabolic regulation and its role in DbCM and lipid metabolism remain unclear. To address this, we utilized cardiomyocyte-specific TGR5-knockout mice to establish DbCM models induced by HFD/STZ and crossed these mice with the genetic diabetic *db/db* mice. Our results demonstrate that TGR5 deletion significantly aggravates myocardial hypertrophy, fibrosis and cardiac dysfunction under diabetic conditions in mice.

Excessive accumulation of fatty acids in the heart leads to lipotoxicity, which contributes to cardiomyocyte dysfunction and pathological remodelling^38^. Thus, the prevention of cardiac lipotoxicity represents a key mechanism to inhibit the development of DbCM. Interestingly, our study revealed a previously unappreciated role of TGR5 in mediating lipid metabolism in cardiomyocytes. TGR5 deficiency significantly contributed to cardiac lipid accumulation in diabetic mice, particularly in FFAs, TGs and DGs, which are known to be cytotoxic lipids. These effects on DbCM and lipid metabolism were also confirmed in TGR5-deficient mice with a *db/db* background and in TGR5-activated *db/db* mice treated with INT-777. These findings suggest that TGR5 plays a protective role in DbCM development by reducing cardiac lipid accumulation and pathological remodelling. In the regulation of calcium signalling, TGR5 operates at the membrane level of cardiomyocyte to alter the metabolic milieu in response to external stimuli, thereby impacting mitochondrial function and calcium homeostasis. This modulation is crucial for the TGR5’s involvement in mitigating myocardial injury. Multiple mechanisms have been proposed to explain ectopic lipid accumulation in the heart, including imbalances in fatty acid uptake, transport, synthesis and consumption, all of which contribute to net lipid storage in the heart. Due to insulin resistance, adipocytes in individuals with diabetes exhibit incomplete inhibition of lipolysis, resulting in increased release of circulating FFAs^39^. This, in turn, enhances cardiac fatty acid uptake and promotes TG storage^40^. Therefore, the absorption of circulating fatty acids into the heart is a crucial regulatory step that influences cardiac lipid content. In our study, TGR5 deletion significantly promoted the uptake of long-chain fatty acids in the heart, while activation of TGR5 using an agonist had the opposite effect. These findings suggest that TGR5 mediates fatty acid uptake and storage in cardiomyocytes of diabetic mice.

Among the three putative groups of fatty acid transporters in cardiac tissue (CD36, FABP-pm and FATPs), CD36 is responsible for up to 60% of fatty acid uptake in the heart^41–43^ and is also associated with the inhibition of fatty acid β-oxidation^44^. CD36 functions as a transmembrane protein, and its localization plays a crucial role in determining its function. Under certain conditions, CD36 translocates to the PM, promoting fatty acid uptake. In our study, we confirmed a significant increase in CD36 expression and the PM localization in cardiomyocytes of mice with DbCM. Unlike macrophages in atherosclerotic mice^9^, TGR5 deletion did not affect the total protein expression of CD36 but significantly enhanced its PM localization in DbCM. Conversely, TGR5 activation impeded the PM localization of CD36. Palmitoylation, a post-translational modification, plays a critical role in regulating the localization and function of CD36. Previous studies have demonstrated that palmitoylation is necessary for CD36 to localize to the PM^45,46^. In hepatocytes, increased CD36 palmitoylation promotes its PM localization, thereby enhancing fatty acid uptake, while inhibition of CD36 palmitoylation reduces fatty acid uptake and promotes fatty acid β-oxidation, participating in the development of non-alcoholic fatty liver disease^44,47^. In our study, we demonstrated that inhibiting CD36 palmitoylation in cardiomyocytes significantly decreased fatty acid uptake and lipid accumulation. Moreover, CD36 palmitoylation was increased in the heart of mice with DbCM, and TGR5 deletion further promoted CD36 palmitoylation and downstream cascades, particularly through the formation of a complex with Fyn and Lyn. Conversely, activation of TGR5 by INT-777 treatment reversed these effects, and were comparable to the effects of a palmitoylation inhibitor. These findings suggest that TGR5 inhibits the PM localization of CD36 by reducing its palmitoylation, ultimately leading to decreased fatty acid uptake. This process re-establishes cellular energetic substrate balance and metabolic homeostasis in the diabetic heart.

Palmitoylation is dynamically catalysed by palmitoyl acyltransferases containing the Asp-His-His-Cys (DHHC) motif. Previous studies have implicated DHHC4, DHHC5 and DHHC6 in the palmitoylation of CD36 (refs. ^29,48^). DHHC4 and DHHC5 are primarily responsible for fatty acid uptake at different stages^29^, while DHHC6 is involved in the uptake of oxidized high-density lipoprotein^25^. DHHC4 and DHHC5 are ubiquitously expressed, with DHHC4 localized in the Golgi apparatus or endoplasmic reticulum for CD36 palmitoylation^49,50^, and DHHC5 primarily localized on the PM to maintain the modification^29,50^. In our study, TGR5 deletion did not affect CD36 palmitoylation and the PM localization under normal conditions. This may be due in part to the lack of PAs, palmitoylation substrates, and the fact that DHHC5 or DHHC6 inhibits DHHC4-mediated CD36 palmitoylation in a compensative manner caused by TGR5 deletion. In addition, we observed that TGR5 deletion enhanced the interaction between CD36 and DHHC4 in cardiomyocytes treated with PA and OA. However, DHHC5 did not exhibit such an interaction. These results were consistent with our findings in cardiomyocytes treated with INT-777. Furthermore, we demonstrated that KD of DHHC4 in cardiomyocytes eliminated the increase in CD36 palmitoylation induced by TGR5 deletion. These results indicate that the TGR5–DHHC4 pathway mediates CD36 palmitoylation in cardiomyocytes, regulating the PM localization of CD36 and lipid uptake.

In summary, our study uncovers a mechanism involved in cardiac fatty acid uptake. The bile acid receptor TGR5 protects against the development of DbCM in response to HFD/STZ consumption or in a genetic model of type 2 diabetes (*db/db* mice). We provide evidence that TGR5 inhibits the PM localization of CD36 by blocking palmitoylation through DHHC4, resulting in reduced fatty acid uptake and lipid accumulation in cardiomyocytes. Furthermore, we observed a significant decrease in the TGR5-biased bile acid DCA in both mice and participants with diabetes complicated by HF, and the plasma DCA levels correlated with cardiac function. These findings highlight the TGR5–DHHC4 pathway as a key mediator of protection against DbCM, and suggest its potential as therapeutic target for intervening in lipid metabolism in DbCM.

## Methods

### Human participants

Human plasma samples were obtained from Fuwai Hospital, Beijing, China. The use of human plasma samples for research purposes was approved by the Institutional Review Board of Fuwai Hospital (2013-496). Written informed consent was obtained from each participant. The participants were divided into two groups: T2DM with LVHT and T2DM with HF. Left ventricular mass index is used to diagnose LVHT as ≥115 g/m^2^ in men and ≥95 g/m^2^ in women. Participants with known aetiology, such as coronary artery disease, hypertension, valvular diseases or hereditary conditions, were excluded. In addition, participants with systemic and infectious diseases, a history of drug or alcohol misuse, or those currently misusing alcohol or drugs were also excluded. Peripheral blood samples were obtained from all participants after overnight fasting. Finally, 48 healthy individuals, 50 individuals with T2DM and LVHTand 42 individuals with T2DM and HF were enrolled in the study. Further details of the participants are listed in Supplementary Table 8.

### Experimental animals

C57BL/6J *Gpbar1*^fl/+^ mice were generated by GemPharmatech using CRISPR–Cas9 technology. C57BKS-*db/+*, C57BKS-*db/db* and αMHC-*cre* mice were purchased from GemPharmatech. αMHC-*Gpbar1*^fl/fl^ (TGR5^*ΔCM*^) mice were generated by crossing *Gpbar1*^fl/+^ and αMHC-*cre* mice. All the mice in the experiments associated with cardiac function, morphology and lipid metabolism were both male and female, and the rest were male only. All mice were housed with ad libitum access to food and water and maintained in a specific pathogen-free animal facility at an ambient temperature of 22 °C and 50% humidity under a 12/12-h light–dark cycle (lights on at 06:00 and lights off at 18:00). The mice were randomly divided into different groups. To assess the nutritional status of the mice, dietary types (normal control diet (NCD) or HFD), body weight, food intake, blood lipid levels and blood glucose levels were recorded in detail. Plasma and heart samples were collected for bile acid profiles after overnight fasting. The age and number of mice used in each experiment are shown in the figure legends. All experimental protocols for the animal studies were approved by the Institutional Animal Care and Use Committee at Peking University Health Science Center (LA2022383) and were carried out in accordance with the National Institutes of Health, Guide for the Care and Use of Laboratory Animals.

### HFD/STZ-induced DbCM in mice

After STZ injection, diabetic mice on the C57BL/6J background were not prone to cardiac functional lesions. Mice that consumed a HFD tended to develop obesity and insulin resistance in the C57BL/6 strain. Therefore, we used a HFD combined with low-dose STZ to establish the DbCM mouse model, as previously described with some modifications^51^. Briefly, insulin resistance was established in TGR5^*ΔCM*^ and TGR5^fl/fl^ mice on a HFD (Research Diets, D12492) for 8 weeks, followed by intraperitoneal injection of 35 mg per kg body weight of STZ (Sigma-Aldrich, S0130) in 50 mmol l^−1^ sodium citrate buffer (pH 4.5) for three consecutive days. The mice were then maintained on a HFD for 16 weeks. Control mice were fed a control diet (Research Diets, D12450J) and administered an intraperitoneal injection of equal amounts of sodium citrate buffer. The HFD was made up of 60% fat, 20% protein and 20% carbohydrate, while the control diet was made up of 10% fat, 20% protein and 70% carbohydrate. Blood glucose and body weight were monitored every 2 weeks. Diabetes was considered successful when random blood glucose levels exceeded 16.7 mmol l^−1^ for two consecutive days. Echocardiography was used to assess cardiac function lesions.

### Generation of TGR5-knockout mice with spontaneous DbCM

*db/db* mice are autosomal recessive models of spontaneous type 2 diabetes derived from C57BL/KsJ inbred strains with leptin receptor gene deficiency and a classic model for studying myocardial complications of type 2 diabetes^52,53^. Cardiomyocyte-specific *Gpbar1*-knockout *db/db* mice were constructed by crossbreeding *db/+* (C57BKS) with αMHC-*Gpbar1*^fl/fl^ (C57BL/6J) mice, followed by mating *db/+;*αMHC-*Gpbar1*^fl/+^ with *db/+;Gpbar1*^fl/+^ mice, to create *db/db;*αMHC-*Gpbar1*^fl/fl^ (*db/db* TGR5^*ΔCM*^) and *db/db;Gpbar1*^fl/fl^ (*db/db* TGR5^fl/fl^) mice. We analysed male *db/db* TGR5^*ΔCM*^, *db/db* TGR5^fl/fl^, and their corresponding control mice in age-matched groups. The *db/db* mice with a mixed C57BKS and C57BL/6J background began to exhibit diabetic symptoms at 4 weeks of age. Blood glucose levels were monitored every 2 weeks. Echocardiography was used to assess cardiac function lesions.

### Treatment of *db/db* mice with INT-777

C57BKS-*db/db* mice aged 20 weeks were treated with 30 mg per kg body weight of INT-777 (MCE, HY-15677) or vehicle (carboxymethyl cellulose) once daily for 12 weeks by gavage. Blood glucose levels were measured every 2 weeks. Echocardiography was used to assess cardiac function lesions. After the experiment, hearts were extracted for further analysis.

### Treatment of HFD/STZ-induced mice with bile acids

Eight-week-old C57BL/6J mice were fed a HFD for 8 weeks, followed by intraperitoneal injection of 35 mg per kg body weight of STZ in 50 mmol l^−1^ sodium citrate buffer (pH 4.5) for three consecutive days. After 12 weeks on this regimen, mice were given DCA (50 mg per kg body weight), TCA (200 mg per kg body weight) or vehicle (carboxymethyl cellulose) once daily for 12 weeks by gavage. Blood glucose levels were measured every 2 weeks. Echocardiography was used to assess cardiac function lesions.

### Fatty acid uptake in vivo

To measure fatty acid uptake in the heart using fluorescent dyes, the mice were prepared^54^. Briefly, each mouse was injected with appropriate doses (0.5 mg per kg body weight) of BODIPY fluorescent-conjugated fatty acids: FL C16 (Invitrogen, D3821), 558/568 C12 (Invitrogen, D3835) and FL C5 (Invitrogen, D3834) in 200 μl of cold HBSS through the tail vein. Thirty minutes after the injection, the hearts were collected, homogenized with RIPA buffer, centrifuged, and the supernatant was extracted for subsequent experiments. Fluorescence intensity (FI) was immediately measured in black 96-well flat-bottom plates using a fluorescence microplate reader. Readings from mice treated with the control solution were used to subtract background signals and were normalized by the weight of the extracted tissue.

### Fatty acid uptake in vitro

To measure fatty acid uptake in cultured cardiomyocytes using fluorescent dyes, BODIPY FL C16 uptake experiments were carried out as previously described with some modifications^54,55^. Primary NMCMs from TGR5-knockout mice or NMCMs treated with INT-777 (30 μmol l^−1^) were incubated in the cardiomyocyte culture medium containing an unlabelled BSA-conjugated fatty acid cocktail (400 μmol l^−1^ PA + 200 μmol l^−1^ OA) for 24 h. The cells were incubated with BODIPY FL C16 for 10 min at 37 °C. After washing thrice with cold PBS, cardiomyocytes were collected using trypsin-EDTA and centrifuged at 600*g* for 3 min at room temperature. The pellet was resuspended in 1 ml cold PBS and centrifuged at 600*g* for 3 min at room temperature. Cardiomyocytes were lysed in 200 μl of PBS and subsequently analysed using a CytoFLEX Flow cytometer (Beckman Coulter). FlowJo (v10.8.1) was used for data analysis.

### Intraperitoneal glucose tolerance test

Intraperitoneal glucose tolerance test was performed^56^. Briefly, mice were fasted for 16 h (from 17:00 to 09:00) and injected intraperitoneally with d-glucose (Sigma, D9434) at 1 g per kg body weight (20% stock, 5 μl per gram). Blood glucose levels were determined via the tail vein at 0, 15, 30, 60, 90 and 120 min after injection.

### Oil Red O and BODIPY lipid staining

Oil Red O and BODIPY 493/503 staining were used to assess the accumulation of neutral lipids in the cardiac tissue and cardiomyocytes. The heart tissue was embedded in OCT compound and subsequently cut into 10-μm-thick sections. For Oil Red O staining, the frozen sections were equilibrated at 37 °C for 30 min and fixed with 4% paraformaldehyde for 10 min at room temperature. After washing with PBS, the sections were placed in 60% isopropyl alcohol for 5 min and covered with an Oil Red O working solution for 15 min (Njjcbio, D027). The sections were sequentially washed thrice with 60% isopropanol and PBS. Images were then visualized under a microscope by NDP.view software (v2.7.52). For BODIPY lipid staining, fixed frozen sections or cardiomyocytes were incubated with 10 μM BODIPY 493/503 (Invitrogen, D3922) for 30 min at room temperature. After washing thrice with PBS, the sections were covered with a mounting medium with DAPI (Abcam, ab104139). The images were visualized using a Zeiss LSM 780 confocal microscope and ZEN-Blue software (v3.7), and were quantified using Image J (v1.8.0) and analysed blindly.

### Isolation of primary NMCMs

Primary cardiomyocytes were isolated and cultured from neonatal wild-type C57BL/6J mice or TGR5-knockout mice^20^. Briefly, the hearts of mice (1-day-old) were harvested and treated using the Neonatal Heart Dissociation Kit (Miltenyi Biotec, 130-098-373). The cells were plated for 1.5 h into 10-cm culture dishes to allow cardiac fibroblast adherence. The suspended cells were collected as cardiomyocytes and cultured in DMEM containing 15% FBS at 37 °C in a humidified 5% CO_2_ incubator for further experiments. Bromodeoxyuridine was added to prevent fibroblast proliferation.

### Immunoprecipitation and western blot

For immunoprecipitation, cell extracts were prepared in NP-40 lysis buffer. The homogenates were immunoprecipitated with an antibody and Pierce Protein A/G Magnetic Beads (Thermo Fisher Scientific, 88802). Bead-conjugated proteins were released using an SDS loading buffer. For western blot, the heart and cells were lysed with RIPA buffer containing a protease/phosphatase inhibitor cocktail (CST, 5872). Total protein concentrations were determined using a Pierce BCA Protein Assay Kit (Thermo, 23225). Protein samples were separated using SDS–PAGE and transferred onto Biotrace nitrocellulose membranes. The blots were blocked for 1 h with 5% non-fat dry milk in 1× TBS/0.5% Tween 20 and incubated overnight at 4 °C with primary antibodies. The blots were incubated for 1 h with secondary antibodies at room temperature, and enhanced chemiluminescence immunodetection was performed by GeneSys software (v1.6.3.0).

### Isolation of plasma membrane protein

The cellular plasma membrane of the heart was isolated using a Minute Kit (Invent Biotechnologies, SM-005) according to the manufacturer’s instructions. Briefly, the heart was homogenized with lysates containing protease inhibitors. To lyse the cells, the homogenates were centrifuged at 16,000*g* for 30 s using a filter. The homogenates were then centrifuged at 700*g* for 1 min to remove the nuclear pellet. The supernatant was subsequently centrifuged at 16,000*g* for 30 min at 4 °C to obtain total membrane protein fractions, including the plasma membrane and organelles. The pellet was resuspended in upper phase and lower phase solutions, followed by centrifugation at 7,800*g* for 15 min at 4 °C to remove organelles. The supernatant was collected, and 1.6 ml of PBS was added to adjust the density of the solution, which was then centrifuged at 16,000*g* for 30 min to save the pellet as plasma membrane proteins. The pellet was redissolved in 50 μl Minute Denaturing Protein Solubilization Reagent (Invent Biotechnologies, WA-009) for immunoblotting.

### Cell line and culture

Human HEK293A cells (SUNNCELL, SNL-247) were used in this study. HEK293A cells were cultured in DMEM medium containing 10% FBS, 100 U ml^−1^ penicillin and 100 mg ml^−1^ streptomycin. Mouse TGR5 and DHHC4 cloned into the expression vector pcDNA3.1 were designed and provided by Shanghai GenePharma. HEK293A cells were transfected with plasmids using Lipofectamine 2000 transfection reagent (Invitrogen, 11668-019).

### TG and FFA contents

Myocardial TG and FFA contents were detected according to the Triglyceride Fluorometric Assay Kit (Elabscience, E-BC-F033) and Free Fatty Acid Assay Kit (Abcam, ab65341), respectively.

### MitoSox staining and 4-HNE assay

For MitoSox staining, the living isolated cardiomyocytes were washed three times with PBS and covered with 5 μmol l^−1^ MitoSox Red probe (MCE, HY-D1055) for 15 min at room temperature. After washing three times with PBS, cells were covered using Hoechst. Images were visualized using a Zeiss LSM 780 confocal microscope and ZEN-Blue software (v3.7). For the 4-HNE assay, the heart tissue was homogenized in pre-cooled PBS and centrifuged at 5,000*g* for 5 min to collect the supernatant. The content of 4-HNE was quantified using a mouse 4-HNE ELISA Kit (FineTest Biotech, EM1583). All samples were normalized to protein content.

### Echocardiography

Mice were anaesthetised with 1–1.5% isoflurane, and transthoracic echocardiography was performed using a 30 MHz MS400 transducer (Vevo 2100 system, Fujifilm VisualSonics)^57^. The parasternal long axis was located in B-mode and the maximum left ventricle length was identified. To measure systolic function, M-mode measurements of the left ventricular internal diameter were obtained in mice under two-dimensional guidance, with a heart rate of >450 beats per min. Left ventricular EF and FS were calculated based on M-mode images. To measure diastolic function, Pulsed-Wave Doppler and Tissue Doppler modes were applied to determine transmitral early (E) wave peak velocities and peak early (E’) annular velocities, respectively. The E/E′ ratio was calculated to evaluate diastolic function. Speckle tracking-based strain analysis (GLS) was performed on the parasternal long axis in B-mode using the Vevo Strain software (v5.7.1).

### Protein palmitoylation analysis

Protein palmitoylation was determined using the IP-ABE method^25^. Briefly, the heart and cells were lysed with RIPA buffer containing protease inhibitors and 50 mM *N*-ethylmaleimide. The homogenates were immunoprecipitated with a CD36 antibody (Novus, NB600-1423; 1:500 dilution) and Pierce Protein A/G Magnetic Beads. The same sample was evenly divided into two parts: one with hydroxylamine treatment (HAM+) and the other without HAM treatment (HAM−). All samples were rotated at room temperature for 1 h to cleave the thioester bonds in the palmitoylated cysteines. Biotin-BMCC was added to the samples, which were then rotated at room temperature for 1 h to specifically label palmitoylated cysteines, followed by immunoblotting. The blots were incubated for 1 h with horseradish peroxidase-conjugated streptavidin (1:10,000 dilution) at room temperature, and then enhanced chemiluminescence immunodetection was performed.

### Measurement of OCR and FAO

The OCR of isolated cells was measured in real time with a Seahorse XFe24 Extracellular Flux Analyzer (Agilent) per the manufacturer’s instructions. The mitochondrial complex inhibitors (oligomycin, FCCP and rotenone/antimycin A) from the XF Cell Mito Stress Test kit (Agilent, 103015-100) were used for both the Seahorse Mito Stress Test and the Seahorse Palmitate Oxidation Stress Test. For the Seahorse Mito Stress Test, the XF DMEM (Agilent, 103575-100) base medium (pH 7.4) was prepared with a glucose solution (1 mol l^−1^, Agilent, 103577-100), pyruvate solution (100 mmol l^−1^, Agilent, 103578-100) and glutamine solution (200 mmol l^−1^, Agilent, 103579-100) before assay. Oligomycin (1.5 μmol l^−1^), FCCP (2 μmol l^−1^) and rotenone/antimycin A (0.5 μmol l^−1^) were injected sequentially. FAO-dependent mitochondrial respiration was measured as oxygen consumption rate differences (∆OCR) with or without palmitate. ∆OCR demonstrated that the preponderance of respiration resulted from FAO. Briefly, the XF DMEM base medium (pH 7.4) was supplemented with 0.5 mmol l^−1^ glucose, 1 mmol l^−1^ glutamine, 0.5 mmol l^−1^ l-carnitine, and 1% FBS (substrate-limited growth media). On the day before the assay, the cells were replaced with the above substrate-limited growth media instead of the cell growth media. The assay media was configured with XF DMEM base medium (pH 7.4) containing 2 mmol l^−1^ glucose and 0.5 mmol l^−1^ l-carnitine. Either palmitate-BSA or BSA control (Agilent, 102720-100) was added immediately before the assay. The analysis by Wave software (v2.6.3) was calibrated by the protein content in each well.

### Bile acid-targeted metabolomics analysis

Bile acid profiles were identified as previously described^17^. Briefly, 30 μl plasma sample was added to 270 μl pre-cooled methanol to precipitate the protein, with 100 nmol l^−1^ deuterated cholic acid-2,2,4,4-D4 (CA-d4) and ursodeoxycholic acid-2,2,4,4-D4 (UDCA-d4) as internal standard. For heart samples, 100 mg tissue was precisely weighed and 900 µl pre-cooled methanol was added to homogenize. Centrifugation was performed to precipitate the protein. After being drained by a vacuum pump, 200 µl methanol was redissolved. The samples were vortexed and then centrifuged to precipitate the particles, and the supernatant was extracted and transferred to an autosampler vial. Samples were measured in an ultra-performance liquid chromatography (UPLC) 100 system coupled to an AB 5600 TripleTOF system (AB SCIEX), with a Waters UPLC CSH C18 column (100 mm × 2.1 mm, 1.7-μm particle size). Multiple reaction monitoring in negative ion mode was used for data acquisition.

### Lipid-targeted metabolomics analysis

Lipid compositions from the hearts or cells were extracted with methyl tert-butyl ether/methanol (5:1 ratio) and spiked with internal standards. The samples were centrifuged, and the supernatants were dried using a vacuum concentrator. The resulting pellet was resuspended in dichloromethane/methanol/H_2_O (60:30:4.5 ratio). The mixed solution was centrifuged, and the supernatant was used for liquid chromatography–tandem mass spectrometry analysis (Biotree Biotech. Co.).

### Statistical analysis

All data were analysed using GraphPad Prism (v9.4.1) and are presented as the mean ± s.e.m. The normal distribution of the data was analysed using the Shapiro–Wilk test. For normally distributed data, differences were compared using a two-tailed Student’s *t*-test for two groups or ANOVA for multiple groups, followed by Tukey’s post hoc test. For data that were not normally distributed, differences were compared using the Mann–Whitney *U* test for two groups or the Kruskal–Wallis test for multiple groups, corrected with Dunn’s analysis. *P* values < 0.05 were considered to indicate statistical significance. No statistical methods were used to predetermine sample sizes, but our sample sizes are similar to those reported in previous publications^17,58^. Data collection and analysis were not performed blind to the conditions of the experiments. No data were excluded from the data analysis.

### Reporting summary

Further information on research design is available in the Nature Portfolio Reporting Summary linked to this article.

## Supplementary information

Supplementary Information Supplementary Figs. 1–4 and Tables 1–10.

Reporting Summary

Supplementary Data 1 Unprocessed gel and western blot images for the supplementary figures.

Supplementary Data 2 All data presented in the graphs for the supplementary figures.

## Source data

Source Data Fig. 1 Statistical source data.

Source Data Fig. 1 Unprocessed western blots.

Source Data Fig. 2 Statistical source data.

Source Data Fig. 3 Statistical source data.

Source Data Fig. 4 Statistical source data.

Source Data Fig. 4 Unprocessed western blots.

Source Data Fig. 5 Statistical source data.

Source Data Fig. 5 Unprocessed western blots.

Source Data Fig. 6 Statistical source data.

Source Data Fig. 6 Unprocessed western blots.

Source Data Fig. 7 Statistical source data.

Source Data Extended Data Fig. 1 Statistical source data.

Source Data Extended Data Fig. 2 Statistical source data.

Source Data Extended Data Fig. 3 Statistical source data.

Source Data Extended Data Fig. 4 Statistical source data.

Source Data Extended Data Fig. 5 Statistical source data.

Source Data Extended Data Fig. 5 Unprocessed western blots.

Source Data Extended Data Fig. 6 Statistical source data.

Source Data Extended Data Fig. 7 Statistical source data.

Source Data Extended Data Fig. 7 Unprocessed western blots.

Source Data Extended Data Fig. 8 Statistical source data.

Source Data Extended Data Fig. 8 Unprocessed western blots.

## Data Availability

All data are available in the paper, Extended Data and Supplementary Information. Source data are provided with this paper.
