# Author Correction: Inhibition of fatty acid uptake by TGR5 prevents diabetic cardiomyopathy

**DOI:** 10.1038/s42255-026-01553-5

**Published:** 2026-06-09

**Authors:** Hu Wang, Jiaxing Wang, Hao Cui, Chenyu Fan, Yuzhou Xue, Huiying Liu, Hui Li, Jianping Li, Houhua Li, Ying Sun, Wengong Wang, Jiangping Song, Changtao Jiang, Ming Xu

**Affiliations:** 1 Department of Cardiology and Institute of Vascular Medicine, Peking University Third Hospital, State Key Laboratory of Vascular Homeostasis and Remodeling, NHC Key Laboratory of Cardiovascular Molecular Biology and Regulatory Peptides, Beijing Key Laboratory of Cardiovascular Receptors Research, Peking University, Beijing, China; 2 State Key Laboratory of Cardiovascular Disease, Fuwai Hospital, National Center for Cardiovascular Diseases, Chinese Academy of Medical Sciences and Peking Union Medical College (CAMS & PUMC), Beijing, China; 3 Department of Physiology and Pathophysiology, School of Basic Medical Sciences, State Key Laboratory of Vascular Homeostasis and Remodeling, Peking University, Beijing, China; 4 Department of Cardiology, Peking University First Hospital, State Key Laboratory of Vascular Homeostasis and Remodeling, Peking University, Beijing, China; 5 State Key Laboratory of Natural and Biomimetic Drugs, Chemical Biology Center, School of Pharmaceutical Sciences, Peking University, Beijing, China; 6 Jiangsu Key Laboratory of New Drug Research and Clinical Pharmacy, Xuzhou Medical University, Xuzhou, China; 7 Department of Biochemistry and Molecular Biology, Beijing Key Laboratory of Protein Posttranslational Modifications and Cell Function, School of Basic Medical Sciences, Peking University Health Science Center, Beijing, China; 8 Center of Basic Medical Research, Institute of Medical Innovation and Research, Peking University Third Hospital, Beijing, China; 9 Research Unit of Medical Science Research Management/Basic and Clinical Research of Metabolic Cardiovascular Diseases, Chinese Academy of Medical Sciences, Beijing, China

**Keywords:** Metabolic diseases, Heart failure, Preclinical research, Heart failure

Correction to: *Nature Metabolism* 10.1038/s42255-024-01036-5, published online 2 May 2024.

In the version of the article initially published, the “C16” control bar in Fig. 4b was incorrect due to a copy–paste error in figure preparation. Fig. 4b and its source data have now been corrected in the HTML and PDF versions of the article, as seen in Fig. 1.

**Fig. 1:**
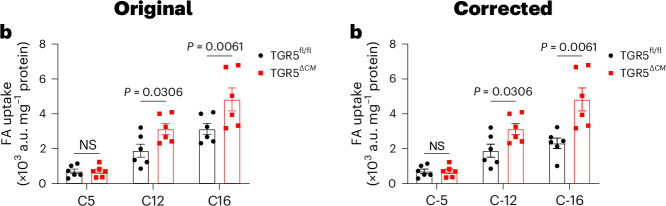
**Original and corrected Fig. 4b.**

