# Supplementary material for: Inhibition of fatty acid uptake by TGR5 prevents diabetic cardiomyopathy
Source: Nat Metab. 2024 May 2;6(6):1161–77. doi: 10.1038/s42255-024-01036-5 (PMC11199146; doi:10.1038/s42255-024-01036-5)
Supplement: Supplementary file 1 — Supplementary Figs. 1–4 and Tables 1–10. [file 42255_2024_1036_MOESM1_ESM.pdf]

nature metabolism

Article

https://doi.org/10.1038/s42255-024-01036-5

Inhibition of fatty acid uptake by TGR5 prevents diabetic cardiomyopathy

In the format provided by the authors and unedited

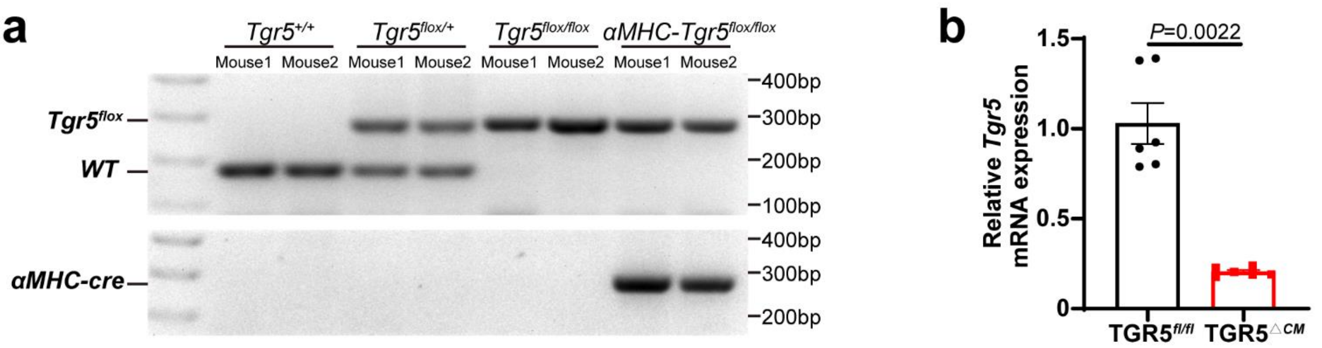

Supplementary Fig. 1 The genotype of *Tgr5*$^{flox/flox}$ mice and validation of cardiomyocyte-specific TGR5 deleted mice. a, Genotyping of *Tgr5*$^{flox/flox}$ mice. **b,** Relative mRNA level of TGR5 in cardiac tissues from TGR5$^{fl/fl}$ and TGR5$^{\triangle CM}$ mice. n = 6. Data are presented as mean ± SEM. Statistical significance was evaluated by two-tailed nonparametric Mann–Whitney test.

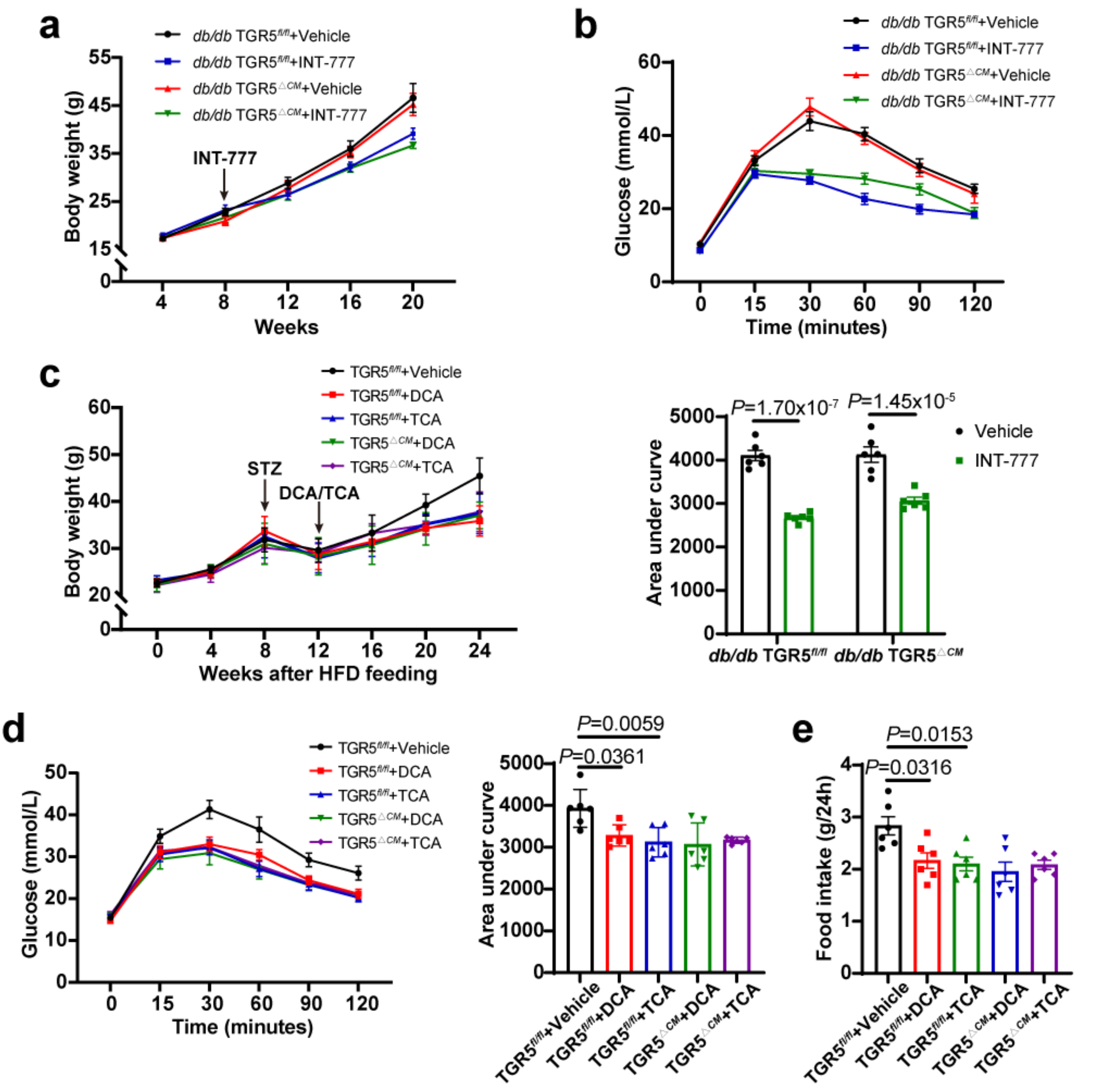

Supplementary Fig. 2 The metabolic phenotype of *db/db* mice treated with INT-777 or HFD/STZ-induced mice treated with bile acids. a, Continuous body weight detection of *db/db* TGR5$^{fl/fl}$ and *db/db* TGR5$^{\triangle CM}$ mice aged 20 weeks with INT-777 treatment or Vehicle for 12 weeks. n = 6. **b,** Intraperitoneal glucose tolerance test of *db/db* TGR5$^{fl/fl}$ and *db/db* TGR5$^{\triangle CM}$ mice treated with INT-777 for 12 weeks (1 g/kg D-glucose). n = 6. **c,** Continuous body weight detection of TGR5$^{fl/fl}$ and TGR5$^{\triangle CM}$ mice challenged with HFD/STZ with DCA or TCA treatment for 12 weeks. n = 6. **d,** Intraperitoneal glucose tolerance test of TGR5$^{fl/fl}$ and TGR5$^{\triangle CM}$ mice challenged with HFD/STZ with DCA or TCA treatment for 12 weeks (1 g/kg D-glucose). n = 6. **e,** 24-hour food intake of TGR5$^{fl/fl}$ and TGR5$^{\triangle CM}$ mice challenged with HFD/STZ with DCA or TCA treatment for 12 weeks. The mice were fasted for 24 h and subsequently exposed to the food. n = 6. Data are presented as mean ± SEM. Statistical significance was evaluated by two-way ANOVA followed by Tukey's post-hoc test (**b**) or one-way ANOVA followed by Tukey's post-hoc test (**d,e**).

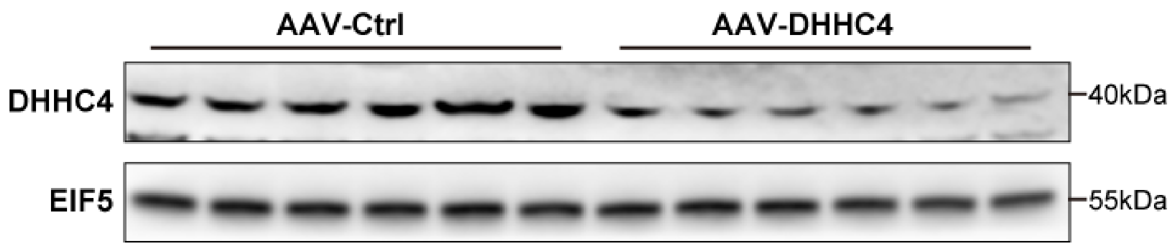

Supplementary Fig. 3 The validation of cardiomyocyte-specific DHHC4 knockdown mice. Representative western blot images of the protein level of DHHC4 in cardiac tissues from mice with AAV-DHHC4 or AAV-Ctrl treatment. The protein level was standardized with that of EIF5. n = 6.

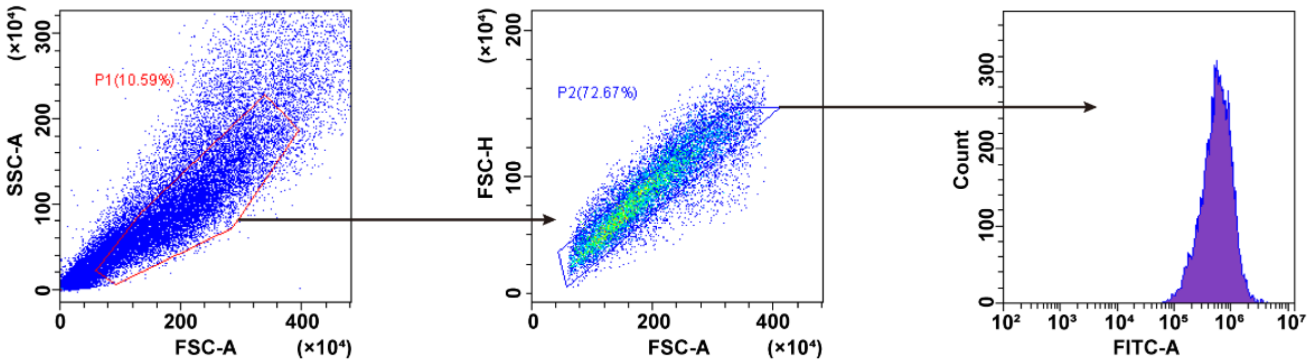

Supplementary Fig. 4 Gating strategy for Flow Cytometry.

	NCD	HFD/STZ
**Unconjugated BA**		
CA (nM)	144.2 (46.7-222.0)	186.8 (36.6-399.0)
CDCA (nM)	13.8 (7.4-24.8)	10.2 (3.1-12.7)
DCA (nM)	24.0 (15.7-96.1)	12.2 (3.8-25.4)*
UDCA (nM)	21.5 (10.7-31.3)	16.6 (12.6-42.5)
LCA (nM)	3.4 (0.6-47.3)	0.3 (0.1-45.0)
αMCA (nM)	47.9 (9.9-75.7)	76.7 (50.6-102.4)
βMCA (nM)	227.4 (74.3-1198.7)	684.2 (150.2-1499.7)
**Conjugated BA**		
GCA (nM)	6.5 (4.7-18.1)	4.8 (1.9-6.5)*
TCA (nM)	616.1 (523.9-1415.6)	393.8 (229.5-611.7)*
TCDCA (nM)	21.6 (8.7-34.6)	19.6 (10.7-25.6)
THDCA (nM)	1.3 (1.1-3.0)	0.8 (0.4-1.4)
TUDCA (nM)	40.6 (18.0-72.9)	16.0 (8.9-35.5)
TDCA (nM)	258.7 (57.4-428.4)	240.5 (86.6-320.3)
TLCA (nM)	0.4 (0.4-0.4)	0.4 (0.4-0.6)
T-αMCA (nM)	117.0 (45.3-255.3)	103.8 (49.8-172.4)
T-βMCA (nM)	71.3 (54.4-699.3)	144.0 (96.8-1057.0)

Supplementary Table 1 Plasma bile acid profiles in mice with normal control diet (NCD) and HFD/STZ-induced diabetic myocardial injury. Data are presented as medians (interquartile ranges). n = 15. $^{*}P<0.05$ versus NCD.

CA: cholic acid; CDCA: chenodeoxycholic acid; DCA: deoxycholic acid; GCA: glycocholic acid; UDCA: ursodeoxycholic acid; TCA: taurocholic acid; TCDCA: taurochenodeoxycholic acid; THDCA: taurohyodeoxycholic acid; TUDCA: tauroursodeoxycholic acid; LCA: lithocholic acid; TDCA: taurodeoxycholic acid; TLCA: taurolithocholic acid; T-αMCA: tauro α-Muricholic acid; T-βMCA: tauro β-Muricholic acid; αMCA: α-Muricholic acid; βMCA: β-Muricholic acid.

	NCD	HFD/STZ
**Unconjugated BA**		
CA (ng/kg)	28.7 (2.6-43.8)	5.4 (1.8-11.4)
CDCA (ng/kg)	6.6 (3.3-18.3)	4.5 (1.2-36.5)
DCA (ng/kg)	78.1 (41.1-112.9)	23.6 (15.0-29.1)*
UDCA (ng/kg)	0.6 (0.4-2.9)	0.3 (0.1-0.9)
LCA (ng/kg)	21.0 (13.8-23.4)	25.1 (16.4-28.1)
αMCA (ng/kg)	8.8 (5.1-21.9)	3.8 (2.8-10.0)
βMCA (ng/kg)	17.3 (3.0-41.2)	6.3 (2.2-23.7)
**Conjugated BA**		
GCA (ng/kg)	0.5 (0.4-0.8)	0.5 (0.2-1.2)
TCA (ng/kg)	45.9 (26.7-69.5)	2.3 (0.2-9.7)*
TCDCA (ng/kg)	4.6 (3.6-8.3)	3.0 (0.9-14.4)
THDCA (ng/kg)	1.2 (0.6-3.6)	1.7 (0.2-8.1)
TUDCA (ng/kg)	2.3 (1.0-5.2)	1.7 (0.5-6.2)
TDCA (ng/kg)	63.0 (27.2-132.6)	49.3 (20.6-243.0)
T-αMCA (ng/kg)	1.9 (0.9-6.9)	1.4 (0.1-11.4)
T-βMCA (ng/kg)	1.8 (0.5-8.5)	0.5 (0.0-30.3)

Supplementary Table 2 Cardiac bile acid profiles in mice with normal NCD and HFD/STZ-induced diabetic myocardial injury. Data are presented as medians (interquartile ranges). n = 15. $^*P<0.05$ versus NCD.

CA: cholic acid; CDCA: chenodeoxycholic acid; DCA: deoxycholic acid; GCA: glycocholic acid; UDCA: ursodeoxycholic acid; TCA: taurocholic acid; TCDCA: taurochenodeoxycholic acid; THDCA: taurohyodeoxycholic acid; TUDCA: tauroursodeoxycholic acid; LCA: lithocholic acid; TDCA: taurodeoxycholic acid; T-αMCA: tauro α-Muricholic acid; T-βMCA: tauro β-Muricholic acid; αMCA: α-Muricholic acid; βMCA: β-Muricholic acid.

	$TGR5^{fl/fl}$		$TGR5^{\triangle CM}$	
	NCD	HFD/STZ	NCD	HFD/STZ
Heart rate (bpm)	488±5	477±14	490±16	475±10
LVAW;d (mm)	0.741±0.024	0.743±0.028	0.721±0.014	0.739±0.014
LVAW;s (mm)	0.922±0.012	0.951±0.031	0.933±0.028	0.919±0.017
LVPW;d (mm)	0.792±0.021	0.857±0.018*	0.796±0.019	0.860±0.014*
LVPW;s (mm)	1.049±0.020	1.040±0.030	1.008±0.019	0.972±0.012
LVID;d (mm)	3.283±0.083	3.632±0.116*	3.107±0.110	3.718±0.092*
LVID;s (mm)	1.885±0.063	2.426±0.086*	1.821±0.098	2.684±0.099*
E (mm/s)	610.123±34.887	615.587±32.016	547.438±20.665	606.867±31.903
A (mm/s)	443.265±20.415	448.968±23.297	384.830±25.434	458.415±21.842
E' (mm/s)	22.301±1.356	17.121±1.069*	20.335±0.990	12.522±0.467*#
A' (mm/s)	19.469±1.167	20.412±1.602	18.801±1.489	17.466±0.896
SV (μ1)	36.347±1.241	26.703±1.456*	34.625±0.839	22.600±0.526*#
CO (ml/min)	12.517±0.661	8.519±0.699*	11.392±1.000	6.363±0.487*#

Supplementary Table 3 Echocardiographic parameters for $TGR5^{fl/fl}$ and $TGR5^{\triangle CM}$ male mice treated with NCD or HFD/STZ. Data are presented as mean ± SEM. n = 10. $^{*}P$<0.05 versus NCD, $^{\#}P$<0.05 versus HFD/STZ $TGR5^{fl/fl}$.

bmp: beats per minutes; LVAW;d: left ventricular end-diastolic anterior wall thickness; LVAW;s: left ventricular end-systolic anterior wall thickness; LVPW;d: left ventricular end-diastolic posterior wall thickness; LVPW;s: left ventricular end-systolic posterior wall thickness; LVID;d: left ventricular end-diastolic diameter; LVID;s: left ventricular end-systolic diameter; SV: stroke volume; CO: cardiac output.

	$TGR5^{fl/fl}$		$TGR5^{\triangle CM}$	
	NCD	HFD/STZ	NCD	HFD/STZ
Heart rate (bpm)	492±10	471±6	493±13	477±18
LVAW;d (mm)	0.691±0.030	0.761±0.011	0.681±0.012	$0.850±0.018^{*\#}$
LVAW;s (mm)	0.858±0.040	0.924±0.022	0.905±0.035	$1.022±0.026^{*\#}$
LVPW;d (mm)	0.676±0.010	$0.780±0.030^{*}$	0.693±0.014	$0.931±0.004^{*\#}$
LVPW;s (mm)	0.953±0.044	1.016±0.023	1.006±0.034	1.076±0.014
LVID;d (mm)	3.320±0.109	3.451±0.113	3.135±0.123	$3.763±0.106^{*}$
LVID;s (mm)	2.020±0.057	$2.395±0.092^{*}$	1.824±0.085	$2.814±0.098^{*\#}$
E (mm/s)	563.188±40.046	561.885±10.274	595.111±30.904	594.403±22.856
A (mm/s)	413.216±18.574	436.113±12.242	469.887±24.430	447.790±29.667
E' (mm/s)	23.280±1.672	$16.517±0.393^{*}$	24.629±2.009	$14.382±1.174^{*}$
A' (mm/s)	22.558±2.397	17.838±0.732	21.580±2.177	16.810±0.954
SV (µ1)	36.638±0.514	$28.325±0.904^{*}$	35.908±0.16	$25.221±0.586^{*}$
CO (ml/min)	14.034±0.368	$11.293±0.514^{*}$	14.205±0.277	$9.071±0.540^{*\#}$

Supplementary Table 4 Echocardiographic parameters for $TGR5^{fl/fl}$ and $TGR5^{\triangle CM}$ female mice treated with NCD or HFD/STZ. Data are presented as mean ± SEM. n = 10. $^{*}P<0.05$ versus NCD, $^{\#}P<0.05$ versus HFD/STZ $TGR5^{fl/fl}$.

bmp: beats per minutes; LVAW;d: left ventricular end-diastolic anterior wall thickness; LVAW;s: left ventricular end-systolic anterior wall thickness; LVPW;d: left ventricular end-diastolic posterior wall thickness; LVPW;s: left ventricular end-systolic posterior wall thickness; LVID;d: left ventricular end-diastolic diameter; LVID;s: left ventricular end-systolic diameter; SV: stroke volume; CO: cardiac output.

	TGR5$^{fl/fl}$		TGR5$^{\triangle CM}$	
	db/m	*db/db*	*db/m*	*db/db*
Heart rate (bpm)	475±6	459±7	480±8	469±13
LVAW;d (mm)	0.544±0.014	0.703±0.028*	0.567±0.025	0.758±0.032*
LVAW;s (mm)	0.633±0.010	0.840±0.036*	0.667±0.009	0.861±0.023*
LVPW;d (mm)	0.623±0.022	0.819±0.019*	0.635±0.019	0.894±0.013$^{*\#}$
LVPW;s (mm)	0.738±0.016	0.977±0.035*	0.727±0.014	1.042±0.020*
LVID;d (mm)	3.054±0.113	3.752±0.087*	3.416±0.117	4.256±0.070$^{*\#}$
LVID;s (mm)	1.834±0.088	2.507±0.077*	2.089±0.089	3.100±0.035$^{*\#}$
E (mm/s)	570.546±42.784	656.694±25.639	584.599±27.933	656.980±16.976
A (mm/s)	441.437±34.591	465.659±31.778	427.041±8.306	394.593±28.962
E' (mm/s)	22.223±1.342	19.531±0.931	22.637±1.412	13.811±0.716$^{*\#}$
A' (mm/s)	19.081±1.208	15.896±1.531	20.223±1.334	19.538±1.133
SV (μl)	40.966±0.629	38.942±1.302	41.581±0.712	28.764±1.533$^{*\#}$
CO (ml/min)	16.296±0.281	12.178±0.487*	16.854±0.550	8.496±0.841$^{*\#}$

Supplementary Table 5 Echocardiographic parameters for TGR5$^{fl/fl}$ and TGR5$^{\triangle CM}$ mice in *db/m* or *db/db* background. Data are presented as mean ± SEM. n = 6. $^{*}P<0.05$ versus *db/m*, $^{\#}P<0.05$ versus *db/db* TGR5$^{fl/fl}$.

bmp: beats per minutes; LVAW;d: left ventricular end-diastolic anterior wall thickness; LVAW;s: left ventricular end-systolic anterior wall thickness; LVPW;d: left ventricular end-diastolic posterior wall thickness; LVPW;s: left ventricular end-systolic posterior wall thickness; LVID;d: left ventricular end-diastolic diameter; LVID;s: left ventricular end-systolic diameter; SV: stroke volume; CO: cardiac output.

	db/db TGR5$^{fl/fl}$		*db/db* TGR5$^{\triangle CM}$	
	Vehicle	INT-777	Vehicle	INT-777
Heart rate (bpm)	460±5	451±4	462±13	476±16
LVAW;d (mm)	0.837±0.028	0.735±0.022*	0.836±0.012	0.844±0.019
LVAW;s (mm)	0.965±0.043	0.879±0.017	0.863±0.016	0.906±0.020
LVPW;d (mm)	0.883±0.017	0.767±0.020*	0.868±0.029	0.863±0.016
LVPW;s (mm)	1.016±0.024	0.966±0.028	0.957±0.034	0.975±0.018
LVID;d (mm)	3.894±0.131	3.417±0.060*	4.099±0.080	4.051±0.174
LVID;s (mm)	2.726±0.126	2.035±0.102*	3.213±0.085	3.047±0.178
E (mm/s)	633.186±26.636	617.262±11.610	653.952±32.586	621.961±34.550
A (mm/s)	422.471±20.611	425.934±27.244	474.571±29.421	401.905±40.939
E' (mm/s)	13.208±0.623	18.102±0.582*	11.168±0.966	10.711±0.503
A' (mm/s)	16.024±0.938	17.650±0.302	17.110±1.353	16.996±1.434
SV (μ1)	32.788±0.920	38.495±1.268*	24.898±1.853	26.566±1.084
CO (ml/min)	10.216±0.477	13.642±0.695*	9.022±0.475	8.697±0.242

Supplementary Table 6 Echocardiographic parameters for *db/db* TGR5$^{fl/fl}$ and *db/db* TGR5$^{\triangle CM}$ mice treated with or without INT-777. Data are presented as mean ± SEM. n = 6. $^{*}P$<0.05 versus Vehicle.

bmp: beats per minutes; LVAW;d: left ventricular end-diastolic anterior wall thickness; LVAW;s: left ventricular end-systolic anterior wall thickness; LVPW;d: left ventricular end-diastolic posterior wall thickness; LVPW;s: left ventricular end-systolic posterior wall thickness; LVID;d: left ventricular end-diastolic diameter; LVID;s: left ventricular end-systolic diameter; SV: stroke volume; CO: cardiac output.

	AAV-Ctrl		AAV-DHHC4	
	TGR5$^{fl/fl}$	TGR5$^{\triangle CM}$	TGR5$^{fl/fl}$	TGR5$^{\triangle CM}$
Heart rate (bpm)	465±8	480±12	463±9	452±9
LVAW;d (mm)	0.799±0.031	0.847±0.013	0.636±0.044*	0.713±0.045*
LVAW;s (mm)	0.889±0.019	0.919±0.026	0.739±0.063*	0.801±0.051
LVPW;d (mm)	0.872±0.032	0.937±0.016	0.736±0.040*	0.768±0.045*
LVPW;s (mm)	1.020±0.022	1.057±0.024	0.875±0.074	0.926±0.065
LVID;d (mm)	3.767±0.139	3.937±0.133	3.209±0.196*	3.611±0.179
LVID;s (mm)	2.647±0.147	3.013±0.097	2.030±0.149*	2.308±0.143*
E (mm/s)	621.504±45.220	629.730±12.974	645.064±72.182	579.344±34.870
A (mm/s)	421.671±42.359	469.903±12.820	421.214±14.717	439.838±29.328
E’ (mm/s)	14.494±0.937	12.147±0.344	20.373±1.733*	18.167±2.096*
A’ (mm/s)	15.939±1.395	15.400±1.684	20.073±2.386	18.362±1.916
SV (μ1)	27.735±0.673	22.810±0.775	35.153±0.812*	32.966±1.014*
CO (ml/min)	11.088±0.337	9.165±0.576	13.583±0.361*	12.605±0.390*

Supplementary Table 7 Echocardiographic parameters for HFD/STZ TGR5$^{fl/fl}$ and TGR5$^{\triangle CM}$ mice treated with or without AAV-DHHC4. Data are presented as mean ± SEM. n = 8. $^{*}P<0.05$ versus AAV-Ctrl.

bmp: beats per minutes; LVAW;d: left ventricular end-diastolic anterior wall thickness; LVAW;s: left ventricular end-systolic anterior wall thickness; LVPW;d: left ventricular end-diastolic posterior wall thickness; LVPW;s: left ventricular end-systolic posterior wall thickness; LVID;d: left ventricular end-diastolic diameter; LVID;s: left ventricular end-systolic diameter; SV: stroke volume; CO: cardiac output.

Groups	Healthy participants (n=48)	T2DM with LVHT (n=50)	T2DM with HF (n=42)
Age, y	50 (44-56)	59 (50-65)	53 (47-60)
Female gender, n (%)	23 (48)	25 (50)	17 (40)
HbA1c (%)	4.60 (4.30-5.20)	6.50 (5.80-7.30)*	6.80 (6.60-7.18)*
TC (mmol/L)	3.94 (3.64-4.34)	3.81 (3.39-4.40)	3.92 (3.22-4.49)
TG (mmol/L)	0.83 (0.64-0.93)	1.27 (0.99-1.90)*	1.18 (0.89-1.69)*
HDL-C (mmol/L)	1.32 (1.14-1.49)	1.07 (0.90-1.32)*	0.83 (0.70-0.99)*#
LDL-C (mmol/L)	1.96 (1.75-2.17)	2.20 (1.71-2.81)	2.51 (1.97-3.01)*
cTnI (ng/mL)	-	0.01 (0.01-0.06)	0.03 (0.02-0.06)
CK-MB (ng/mL)	-	1.35 (0.85-2.04)	1.80 (1.13-2.43)
NT-proBNP (pg/mL)	-	851.75 (276.08-1881.25)	2854.00 (1524.75-4797.25)#
LVEF (%)	-	60.50 (56.00-65.00)	25.00 (22.25-28.00)#
**Male**			
Age, y	46 (40-50)	59 (46-65)	53 (48-60)
HbA1c (%)	4.60 (4.30-5.20)	6.65 (5.98-7.15)*	6.70 (6.50-7.10)*
TC (mmol/L)	3.98 (3.66-4.38)	3.72 (3.36-4.09)	3.94 (3.47-4.37)
TG (mmol/L)	0.82 (0.64-0.94)	1.38 (0.94-1.91)*	1.22 (0.89-2.01)*
HDL-C (mmol/L)	1.30 (1.18-1.39)	1.01 (0.83-1.15)*	0.81(0.70-0.94)#
LDL-C (mmol/L)	1.95 (1.67-2.15)	2.12 (1.58-2.37)	2.46 (2.19-2.98)*
cTnI (ng/mL)	-	0.02 (0.01-0.06)	0.03 (0.02-0.06)
CK-MB (ng/mL)	-	1.30 (0.98-2.13)	1.93 (1.50-2.43)
NT-proBNP (pg/mL)	-	527.40 (210.00-1069.00)	3255.00 (1670.00-4889.00)#
LVEF (%)	-	60.00 (55.00-65.00)	25.00 (23.00-28.00)#
**Female**			
Age, y	55 (49-58)	58 (54-65)	50 (45-57)
HbA1c (%)	4.60 (4.40-5.50)	6.40 (5.70-7.50)*	6.80 (6.70-7.30)*
TC (mmol/L)	3.94 (3.45-4.32)	4.20 (3.49-4.78)	3.80 (2.99-4.60)
TG (mmol/L)	0.83 (0.64-0.92)	1.21 (1.00-1.46)*	1.07 (0.89-1.51)*
HDL-C (mmol/L)	1.32 (1.10-1.56)	1.15 (0.97-1.36)	0.89 (0.69-1.24)*#
LDL-C (mmol/L)	1.99 (1.79-2.29)	2.38 (1.91-3.13)	2.60 (1.90-3.02)
cTnI (ng/mL)	-	0.01 (0.01-0.05)	0.03 (0.01-0.10)
CK-MB (ng/mL)	-	1.53 (0.82-2.03)	1.35 (0.98-2.36)
NT-proBNP (pg/mL)	-	1191.00 (358.00-2423.00)	2617.00 (1316.00-3917.00)#
LVEF (%)	-	61.00 (56.00-65.00)	26.00 (21.00-27.00)#

Supplementary Table 8 Characteristics of included study participants. HbA1c: glycated hemoglobin; TC: total cholesterol; TG: triglyceride; HDL-C: high-density lipoprotein cholesterol; LDL-C: low-density lipoprotein cholesterol; cTnI: cardiac troponin; CK-MB: creatine kinase isoenzymes; NT-proBNP: N-terminal pro-brain natriuretic peptide; LVEF: left ventricular ejection fractions. Data are presented as n (%) for gender and medians (interquartile ranges) for other clinical indicators. $^{*}P<0.05$ versus healthy subjects, $^{\#}P<0.05$ versus T2DM with LVHT.

	Healthy participants	T2DM with LVHT	T2DM with HF
**Unconjugated BA**			
CA (nM)	85.9 (54.6-171.5)	54.4 (30.0-117.9)	72.2 (20.3-121.0)
CDCA (nM)	155.7 (104.9-243.8)	153.2 (29.5-232.1)	70.3 (5.9-161.1)
DCA (nM)	450.5 (248.3-690.0)	179.6 (119.7-338.0)*	70.2 (32.7-124.5)*#
UDCA (nM)	53.7 (32.4-91.5)	41.3 (13.5-114.8)	33.0 (20.4-63.1)
LCA (nM)	28.0 (14.6-35.8)	22.2 (13.1-34.4)	18.7 (11.4-31.7)
**Conjugated BA**			
GCA (nM)	304.0 (120.7-803.0)	188.0 (103.0-391.4)	161.7 (43.2-235.3)*
GUDCA (nM)	136.9 (76.1-252.6)	102.6 (31.5-253.3)	110.1 (32.2-723.8)
GCDCA (nM)	719.8 (369.2-1226.4)	520.8 (252.1-773.3)	445.8 (278.1-916.0)
GDCA (nM)	895.3 (402.3-1593.6)	627.6 (130.5-2246.1)	463.3 (124.2-3220.4)
GLCA (nM)	44.2 (22.7-109.1)	51.3 (24.0-67.8)	50.4 (28.0-130.7)
TCA (nM)	62.5 (20.2-113.3)	23.1 (12.6-46.9)*	16.5 (9.3-23.9)*
TCDCA (nM)	61.5 (18.6-89.1)	28.0 (16.2-57.0)	29.4 (14.8-56.8)
THDCA (nM)	2.9 (1.3-4.6)	1.3 (0.6-3.1)	1.5 (0.5-3.7)
TUDCA (nM)	10.9 (5.3-24.2)	7.7 (5.2-16.0)	8.2 (3.4-19.0)
TDCA (nM)	20.2 (5.7-86.2)	12.4 (5.0-20.6)	16.2 (5.3-49.0)
TLCA (nM)	1.8 (0.9-3.1)	1.4 (0.9-2.6)	1.8 (1.0-3.2)
**Male**			
**Unconjugated BA**			
CA (nM)	96.4 (75.0-174.1)	79.7 (30.0-164.3)	77.7 (36.9-153.6)
CDCA (nM)	186.5 (117.4-256.9)	142.0 (49.9-192.2)	54.6 (4.1-155.6)
DCA (nM)	560.4 (285.1-649.4)	163.0 (124.8-256.7)*	90.4 (33.8-132.4)*#
UDCA (nM)	53.6 (18.9-124.7)	44.5 (16.6-114.2)	27.9 (11.3-69.4)
LCA (nM)	31.5 (14.1-39.9)	16.4 (12.8-31.9)	14.1 (11.7-21.3)
**Conjugated BA**			
GCA (nM)	401.3 (104.4-820.7)	171.7 (102.1-463.2)	144.5 (15.5-253.4)*
GUDCA (nM)	135.5 (62.0-248.8)	119.2 (26.6-515.5)	112.7 (30.3-369.3)
GCDCA (nM)	642.3 (304.8-1270.0)	506.0 (222.7-878.6)	666.4 (375.3-1250.9)
GDCA (nM)	1012.5 (382.0-2617.9)	748.9 (315.5-2478.5)	1065.7 (221.5-3871.7)
GLCA (nM)	41.9 (20.3-109.1)	59.5 (24.0-69.0)	60.4 (28.6-107.0)
TCA (nM)	71.6 (22.9-128.8)	25.1 (16.9-58.1)	12.5 (7.8-19.3)*
TCDCA (nM)	58.9 (19.6-85.8)	31.5 (10.5-52.0)	34.6 (16.3-78.6)
THDCA (nM)	2.9 (1.2-4.8)	1.5 (0.7-3.3)	1.6 (0.3-3.9)
TUDCA (nM)	8.9 (5.2-18.0)	6.4 (5.2-21.1)	7.4 (3.8-18.8)
TDCA (nM)	11.2 (4.6-87.7)	11.1 (6.4-20.4)	9.3 (5.4-43.3)
TLCA (nM)	1.6 (0.9-2.5)	1.6 (0.9-2.5)	2.1 (1.4-3.2)
**Female**			
**Unconjugated BA**			
CA (nM)	55.0 (41.8-143.5)	47.5 (27.6-108.3)	30.0 (13.3-87.8)
CDCA (nM)	144.4 (90.4-208.1)	178.6 (18.6-281.9)	110.1 (30.3-200.5)
DCA (nM)	346.0 (230.5-697.2)	232.8 (87.8-344.0)*	62.0 (27.4-87.2)*#
UDCA (nM)	53.7 (38.9-84.2)	31.1 (10.6-115.0)	35.5 (27.3-51.3)
LCA (nM)	26.9 (16.2-33.6)	31.2 (13.8-35.7)	29.1 (11.3-34.6)
**Conjugated BA**			
GCA (nM)	217.4 (124.4-649.2)	196.9 (106.9-263.2)	174.7 (105.7-228.8)
GUDCA (nM)	138.3 (84.1-276.7)	93.3 (44.0-187.7)	107.5 (42.7-861.8)

GCDCA (nM)	736.9 (481.1-1064.1)	535.6 (302.7-716.4)	419.6 (273.2-567.8)
GDCA (nM)	822.1 (469.5-1363.7)	426.4 (90.4-1884.8)	159.6 (87.1-2175.5)
GLCA (nM)	46.8 (22.9-109.1)	32.8 (27.3-56.2)	43.2 (23.3-172.0)
TCA (nM)	54.1 (20.3-109.9)	15.9 (12.2-32.2)*	21.6 (18.4-36.1)
TCDCA (nM)	61.5 (18.3-150.4)	25.8 (20.3-77.2)	25.3 (14.1-49.0)
THDCA (nM)	3.0 (1.5-4.5)	1.2 (0.7-2.1)	1.5 (0.8-3.1)
TUDCA (nM)	18.4 (5.4-35.0)	11.1 (4.8-14.2)	12.6 (3.4-49.8)
TDCA (nM)	28.9 (10.9-81.6)	16.7 (4.2-26.1)	18.0 (5.3-44.5)
TLCA (nM)	2.3 (1.3-3.5)	1.4 (0.8-2.5)	1.2 (0.7-3.3)

Supplementary Table 9 Bile acid profiles in collected clinical plasma samples from healthy participants (n = 48), T2DM with LVHT (n = 50) and T2DM with HF (n = 42). Data are presented as medians (interquartile ranges). $^{*}P<0.05$ versus healthy participants, $^{\#}P<0.05$ versus T2DM with LVHT.

CA: cholic acid; CDCA: chenodeoxycholic acid; DCA: deoxycholic acid; UDCA: ursodeoxycholic acid; LCA: lithocholic acid; GCA: glycocholic acid; GUDCA: glycoursodeoxycholic acid; GCDCA: glycochenodeoxycholic acid; GDCA: glycodeoxycholic acid; GLCA: glycolithocholic acid; TCA: taurocholic acid; TCDCA: taurochenodeoxycholic acid; THDCA: taurohyodeoxycholic acid; TUDCA: tauroursodeoxycholic acid; TDCA: taurodeoxycholic acid; TLCA: taurolithocholic acid.

**Name**	**Sequences of oligonucleotides (5’→3’)**	
	**Forward**	**Reverse**
(A) Primers for mouse Generation and GenotypinG		
Tgr5^{flox}	5’-CATGCTTGTAGGGTGATGTGACAG-3’	5’-TGTGCTGAAGCAAGGTCTCACTC-3’
αMHC-*cre*	5’-ATGACAGACAGATCCCTCCTATCTCC-3’	5’-CTCATCACTCGTTGCATCATCGAC-3’
(B) shRNA tarGetinG sequence (mouse)		
Dhhc4	5’-CCACAAATGAATGGTATAAAG-3’	5’-CTTTATACCATTCATTTGTGG-3’
(C) siRNA tarGetinG sequence (mouse)		
Dhhc4	5’-UUCCUACUGGGCUUUGUCATT-3’	5’-UGACAAAGCCCAGUAGGAATT-3’
(D) qPCR Primers (mouse)		
Tgr5	5’-TCCTGTCAGTCTTGGCCTATGA-3’	5’-GGTGCTGCCCAATGAGATG-3’
18s	5’-GTAACCCGTTGAACCCATT-3’	5’-CCATCCAATCGGTAGTAGCG-3’

Supplementary Table 10 Sequences of oligonucleotides.
