# Supplementary material for: Inhibition of fatty acid uptake by TGR5 prevents diabetic cardiomyopathy
Source: Nat Metab. 2024 May 2;6(6):1161–77. doi: 10.1038/s42255-024-01036-5 (PMC11199146; doi:10.1038/s42255-024-01036-5)
Supplement: Supplementary file 2 — Reporting Summary [file 42255_2024_1036_MOESM2_ESM.pdf]

nature portfolio

Corresponding author(s): Ming Xu

Last updated by author(s): Mar 5, 2024

Reporting Summary

Nature Portfolio wishes to improve the reproducibility of the work that we publish. This form provides structure for consistency and transparency in reporting. For further information on Nature Portfolio policies, see our Editorial Policies and the Editorial Policy Checklist.

Statistics

For all statistical analyses, confirm that the following items are present in the figure legend, table legend, main text, or Methods section.

n/a	Confirmed	
☐	☒	The exact sample size (*n*) for each experimental group/condition, given as a discrete number and unit of measurement
☐	☒	A statement on whether measurements were taken from distinct samples or whether the same sample was measured repeatedly
☐	☒	The statistical test(s) used AND whether they are one- or two-sided<br>*Only common tests should be described solely by name; describe more complex techniques in the Methods section.*
☒	☐	A description of all covariates tested
☐	☒	A description of any assumptions or corrections, such as tests of normality and adjustment for multiple comparisons
☐	☒	A full description of the statistical parameters including central tendency (e.g. means) or other basic estimates (e.g. regression coefficient) AND variation (e.g. standard deviation) or associated estimates of uncertainty (e.g. confidence intervals)
☐	☒	For null hypothesis testing, the test statistic (e.g. *F*, *t*, *r*) with confidence intervals, effect sizes, degrees of freedom and *P* value noted<br>*Give P values as exact values whenever suitable.*
☒	☐	For Bayesian analysis, information on the choice of priors and Markov chain Monte Carlo settings
☒	☐	For hierarchical and complex designs, identification of the appropriate level for tests and full reporting of outcomes
☒	☐	Estimates of effect sizes (e.g. Cohen's *d*, Pearson's *r*), indicating how they were calculated

Our web collection on statistics for biologists contains articles on many of the points above.

Software and code

Policy information about availability of computer code

Data collection	ZEN-Blue (v3.7) imaging software were used to acquire confocal images.<br>NDP.view software (v2.7.52) was used to obtain HE, Masson and Oil Red O images.<br>GeneSys software (v1.6.3.0) was used to acquire Western blot images and Image J for Western blot quantification.<br>XFe24 Extracellular Flux Analyzer was used to Seahorse analysis.<br>CytoFLEX Flow cytometer (Beckman Coulter).<br>Vevo 2100 system (Fujifilm VisualSonics) was used to perform echocardiography.<br>Excel 2021 was used for data collection.
Data analysis	GraphPad Prism (v9.4.1).<br>Image J software program (v1.8.0).<br>FlowJo (v10.8.1).<br>CytExpert (v2.5.0.77).<br>Wave (v2.6.3)<br>Vevo Strain Software (v5.7.1)

For manuscripts utilizing custom algorithms or software that are central to the research but not yet described in published literature, software must be made available to editors and reviewers. We strongly encourage code deposition in a community repository (e.g. GitHub). See the Nature Portfolio guidelines for submitting code & software for further information.

Data

Policy information about availability of data

All manuscripts must include a data availability statement. This statement should provide the following information, where applicable:

- Accession codes, unique identifiers, or web links for publicly available datasets
- A description of any restrictions on data availability
- For clinical datasets or third party data, please ensure that the statement adheres to our policy

Source data are provided with this paper. All other data are available in the manuscript, extended data and supplementary information. Additional source data and other findings of this study are available from the corresponding authors.

Research involving human participants, their data, or biological material

Policy information about studies with human participants or human data. See also policy information about sex, gender (identity/presentation), and sexual orientation and race, ethnicity and racism.

Reporting on sex and gender	48 healthy subjects, 50 T2DM with LVHT and 42 T2DM with HF were enrolled in the study, which include 75 male and 65 female. Sex of participants was determined based on self-report. The detailed information of subjects is listed in Supplementary Table 8.
Reporting on race, ethnicity, or other socially relevant groupings	All the participants were Chinese and belong to the Mongolian race.
Population characteristics	A detailed table with characteristics of the study population is provided in Supplementary Table 8 of this work.
Recruitment	Human plasma samples were obtained from Fuwai Hospital, Beijing, China. Written informed consent was obtained from each participant. The patients were divided into two groups: T2DM with LVHT and T2DM with HF. LV mass index (LVMI) is used to diagnose LVHT as ≥115 g/m2 in men and ≥95 g/m2 in women. Patients with known etiology, such as coronary artery disease, hypertension, valvular diseases or hereditary conditions, were excluded. In addition, participants with systemic and infectious diseases, the history of drug or alcohol abuse, or currently abusing alcohol or drugs were also excluded.
Ethics oversight	The use of human plasma samples for research purposes was approved by the Institutional Review Board of Fuwai Hospital (2013-496).

Note that full information on the approval of the study protocol must also be provided in the manuscript.

Field-specific reporting

Please select the one below that is the best fit for your research. If you are not sure, read the appropriate sections before making your selection.

☒ Life sciences ☐ Behavioural & social sciences ☐ Ecological, evolutionary & environmental sciences

For a reference copy of the document with all sections, see nature.com/documents/nr-reporting-summary-flat.pdf

Life sciences study design

All studies must disclose on these points even when the disclosure is negative.

Sample size	No statistical methods were used to predetermine sample size (n). Number of sample was determined based on experimental approach, availability, feasibility required to obtain definitive results. All "n" values have been provided in the figure legends.
Data exclusions	No data were excluded.
Replication	All of experiments have been successfully repeated more than twice and/or with sufficient cells/animals per group to demonstrate statistical significance and similar results were obtained. All experiments were statistically analyzed.
Randomization	The mice were randomly assigned to each experimental/control group. For cell culture experiments, individual wells were randomized into groups of treatment conditions.
Blinding	Investigators were blinded during manual cell counting and imaging analysis. Collection of raw data from animals (eg. body weight, blood glucose) and tissue harvesting for analyses was not blinded because it was necessary to preserve treatment information of each animal.

Reporting for specific materials, systems and methods

We require information from authors about some types of materials, experimental systems and methods used in many studies. Here, indicate whether each material, system or method listed is relevant to your study. If you are not sure if a list item applies to your research, read the appropriate section before selecting a response.

Materials & experimental systems

n/a	Involved in the study
☐	☒ Antibodies
☐	☒ Eukaryotic cell lines
☒	☐ Palaeontology and archaeology
☐	☒ Animals and other organisms
☒	☐ Clinical data
☒	☐ Dual use research of concern
☒	☐ Plants

Methods

n/a	Involved in the study
☒	☐ ChIP-seq
☐	☒ Flow cytometry
☒	☐ MRI-based neuroimaging

Antibodies

Antibodies used

The following antibodies were used: CD36 (Abcam; cat#: ab124515; dilution: 1:1000 for WB), β-MHC (ABclonal; cat#: A22140; dilution: 1:1000 for WB), ANP (Abcam; cat#: ab225844; dilution: 1:1000 for WB), BNP (ABclonal; cat#: A2179; dilution: 1:1000 for WB), Na, K-ATPase (Abcam; cat#: ab76020; dilution: 1:1000 for WB), DHHC4 (Abcam; cat#: ab235369; dilution: 1:1000 for WB), DHHC5 (Sigma; cat#: HPA014670; dilution: 1:1000 for WB), Fyn (CST; cat#: 4023S; dilution: 1:2000 for WB), Lyn (CST; cat#: 2796S; dilution: 1:2000 for WB), p-JNK (CST; cat#: 9251S; dilution: 1:2000 for WB), JNK (CST; cat#: 9252S; dilution: 1:2000 for WB), GST (CST; cat#: 2625S; dilution: 1:2000 for WB), p-PKA substrate (CST; cat#: 9624S; dilution: 1:1000 for WB), PKA (CST; cat#: 4782S; dilution: 1:1000 for WB), GAPDH (CST; cat#: 2118S; dilution: 1:5000 for WB), EIF5 (Santa cruz; cat#: sc-28309; dilution: 1:1000 for WB), HRP streptavidin (Proteintech; cat#: SA00001-0; dilution: 1:10000 for WB), HRP anti-Rabbit IgG (H+L) (ZSGB-BIO; cat#: ZB-2301; dilution: 1:5000 for WB) and HRP anti-Mouse IgG (H+L) (ZSGB-BIO; cat#: ZB-2305; dilution: 1:5000 for WB) for western blot. CD36 (Novus; cat#: NB600-1423; dilution: 1:200 for IF), Na, K-ATPase (Abcam; cat#: ab7671; dilution: 1:200 for IF), Anti-Rabbit IgG (H+L) 488 (Invitrogen; cat#: A21206; dilution: 1:500 for IF) and Anti-Mouse IgG (H+L) 568 (Invitrogen; cat#: a10037; dilution: 1:500 for IF) for Immunofluorescence staining.

Validation

All antibodies were obtained from commercial vendors and the validity can be retrieved from the manufacturer' website or widely used by other researchers. We further made sure to identify the correct weight or expression localization for each of the antibodies.
CD36 (Abcam, ab124515): https://www.abcam.cn/products/primary-antibodies/cd36-antibody-ab124515.html
β-MHC (ABclonal, A22140): https://abclonal.com.cn/catalog/A22140
ANP (Abcam, ab225844): https://www.abcam.cn/products/primary-antibodies/natriuretic-peptides-a-antibody-epr22089-283-ab225844.html
BNP (ABclonal, A2179): https://abclonal.com.cn/catalog/A2179
Na, K-ATPase (Abcam, ab76020): https://www.abcam.cn/products/primary-antibodies/sodium-potassium-atpase-antibody-ep1845y-plasma-membrane-loading-control-ab76020.html
DHHC4 (Abcam, ab235369): https://www.abcam.cn/products/primary-antibodies/zdhhc4dc1-antibody-ab235369.html
DHHC5 (Sigma, HPA014670): https://www.sigmaaldrich.cn/CN/zh/search/hpa014670?focus=products&page=1&perpage=30&sort=relevance&term=hpa014670&type=product
Fyn (CST, 4023S): https://www.cellsignal.cn/products/primary-antibodies/fyn-antibody/4023;jsessionid=gc6dn8prlhddddtl-a_noxlzuanbburiwyl9ydol.prod_store01?N=4294956287&Ntt=4023s&_requestid=2384274&fromPage=plp&site-search-type=Products
Lyn (CST, 2796S): https://www.cellsignal.cn/products/primary-antibodies/lyn-c13f9-rabbit-mab/2796?site-search-type=Products&N=4294956287&Ntt=2796s&fromPage=plp&_requestid=2384416
p-JNK (CST, 9251S): https://www.cellsignal.cn/products/primary-antibodies/phospho-sapk-jnk-thr183-tyr185-antibody/9251?site-search-type=Products&N=4294956287&Ntt=9251s&fromPage=plp&_requestid=2384508
JNK (CST, 9252S): https://www.cellsignal.cn/products/primary-antibodies/sapk-jnk-antibody/9252?site-search-type=Products&N=4294956287&Ntt=9252s&fromPage=plp&_requestid=2384569
GST (CST, 2625S): https://www.cellsignal.cn/products/primary-antibodies/gst-tag-91g1-rabbit-mab/2625?site-search-type=Products&N=4294956287&Ntt=2625s&fromPage=plp&_requestid=2384632
p-PKA substrate (CST, 9624S): https://www.cellsignal.cn/products/primary-antibodies/phospho-pka-substrate-rrxs-t-100g7e-rabbit-mab/9624?site-search-type=Products&N=4294956287&Ntt=9624s&fromPage=plp&_requestid=2384704
PKA (CST, 4782S): https://www.cellsignal.cn/products/primary-antibodies/pka-c-a-antibody/4782?site-search-type=Products&N=4294956287&Ntt=4782s&fromPage=plp&_requestid=2384783
GAPDH (CST, 2118S): https://www.cellsignal.cn/products/primary-antibodies/gapdh-14c10-rabbit-mab/2118?site-search-type=Products&N=4294956287&Ntt=2118s&fromPage=plp&_requestid=2384854
EIF5 (Santa cruz, sc-28309): https://www.scbt.com/p/eif5-antibody-e-10?requestFrom=search
HRP streptavidin (Proteintech, SA00001-0): https://www.ptgcn.com/products/HRP-conjugated-Streptavidin-secondary-antibody.htm
HRP anti-Rabbit IgG (H+L) (ZSGB-BIO, ZB-2301): http://www.zsbio.com/search?q=ZB-2301
HRP anti-Mouse IgG (H+L) (ZSGB-BIO, ZB-2305): http://www.zsbio.com/search?q=ZB-2305
CD36 (Novus, NB600-1423): https://www.novusbio.com/products/cd36-antibody-fa6-152_nb600-1423
Na, K-ATPase (Abcam, ab7671):
https://www.abcam.cn/products/primary-antibodies/alpha-1-sodium-potassium-atpase-antibody-4646-ab7671.html
Anti-Rabbit IgG (H+L) 488 (Invitrogen, A21206): https://www.thermofisher.cn/cn/zh/antibody/product/Donkey-anti-Rabbit-IgG-H-L-Highly-Cross-Adsorbed-Secondary-Antibody-Polyclonal/A-21206
Anti-Mouse IgG (H+L) 568 (Invitrogen, A10037): https://www.thermofisher.cn/cn/zh/antibody/product/Donkey-anti-Mouse-IgG-H-L-Highly-Cross-Adsorbed-Secondary-Antibody-Polyclonal/A10037

Eukaryotic cell lines

Policy information about cell lines and Sex and Gender in Research

Cell line source(s)	HEK293A cells (SUNNCELL, SNL-247, female) were used in this study.
Authentication	HEK293A was not specifically authenticated beyond being obtained from SUNNCELL.
Mycoplasma contamination	Mycoplasma detection was performed in accordance with department protocols and tested negative.
Commonly misidentified lines (See ICLAC register)	No commonly misidentified lines were used.

Animals and other research organisms

Policy information about studies involving animals; ARRIVE guidelines recommended for reporting animal research, and Sex and Gender in Research

Laboratory animals	C57BL/6J Tgr5fl/+ mice were generated by GemPharmatech using CRISPR/Cas9 technology. C57BKS-db/+, C57BKS-db/db, and αMHC-cre mice were purchased from GemPharmatech. αMHC-Tgr5fl/fl (TGR5△CM) mice were generated by crossing Tgr5fl/+ and αMHC-cre mice. All of the mice in the experiments associated with cardiac function, morphology, and lipid metabolism were male and female, and the rest were male only. All mice were housed with ad libitum access to food and water and maintained in a specific pathogen-free animal facility at an ambient temperature of 22? and 50% humidity under a 12/12 h light/dark cycle (lights on at 6:00 a.m. and lights off at 6:00 p.m.). To assess the nutritional status of the mice, dietary types (control diet or HFD), body weight and food intake of the mice, as well as blood lipid and blood glucose levels were recorded in detail. Plasma and heart from mice after overnight fasting were collected for bile acid profiles. The age and number of mice used in each experiment are shown in the figure legends.
Wild animals	The study did not involve wild animals.
Reporting on sex	Experimental findings in this study apply to both male and female animals. As both sexes were used for the repetition of key experiments in vivo, the research conclusions are applicable to both sexes.
Field-collected samples	The study did not include samples collected from the field.
Ethics oversight	All experimental protocols for the animal studies were approved by the Institutional Animal Care and Use Committee at Peking University Health Science Center (LA2022383).

Note that full information on the approval of the study protocol must also be provided in the manuscript.

Flow Cytometry

Plots

Confirm that:

☒ The axis labels state the marker and fluorochrome used (e.g. CD4-FITC).

☒ The axis scales are clearly visible. Include numbers along axes only for bottom left plot of group (a 'group' is an analysis of identical markers).

☒ All plots are contour plots with outliers or pseudocolor plots.

☒ A numerical value for number of cells or percentage (with statistics) is provided.

Methodology

Sample preparation	To measure fatty acid uptake in cultured cardiomyocytes using fluorescent dyes, BODIPY FL C16 uptake experiments were carried out. Primary NMCMs from TGR5 knockout mice or NMCMs treated with INT-777 (30 μmol/L) were incubated in the cardiomyocyte culture medium containing an unlabeled BSA-conjugated fatty acid cocktail (400 μmol/L PA + 200 μmol/L OA) for 24 h. The cells were incubated with BODIPY FL C16 (a fluorescent dye) for 10 min at 37 °C. After washing thrice with cold PBS, cardiomyocytes were collected using trypsin-EDTA and centrifuged at 600 × g for 3 min at room temperature. The pellet was resuspended in 1 mL cold PBS and centrifuged at 600 × g for 3 min at room temperature. Cardiomyocytes were lysed in 200 μL of PBS and subsequently analyzed using flow cytometry.
Instrument	CytoFLEX S Flow cytometer (Beckman Coulter)
Software	CytExpert (v2.5.0.77)
Cell population abundance	In flow analysis, we initially recorded 10,000 events representing the total cell population. Our initial gating strategy was based on FSC-A/SSC-A, we retained 10.59% of the total events (12,471 cells).To isolate single cells, we applied a gating strategy using FSC-A/FSC-H, leaving us with 9,063 cells, 72.67% of the cells passing the preceding FSC-A/SSC-A gate and 7.70% of the total events. A total of 9,063 cells were used for the final analysis.

Gating strategy	The first gate was applied based on the FSC parameter, which generally corresponds to cell size, and SSC parameter reflects cellular granularity or complexity. This allowed us to exclude debris and select total cell population. The second gate was based on FSC-A/FSC-H, which further refined our single-cell population. Finally, we present our results with a histogram.

☒ Tick this box to confirm that a figure exemplifying the gating strategy is provided in the Supplementary Information.
