# Supplementary figures and images for: Inhibition of fatty acid uptake by TGR5 prevents diabetic cardiomyopathy
Source: Nat Metab. 2024 May 2;6(6):1161–77. doi: 10.1038/s42255-024-01036-5 (PMC11199146; doi:10.1038/s42255-024-01036-5)

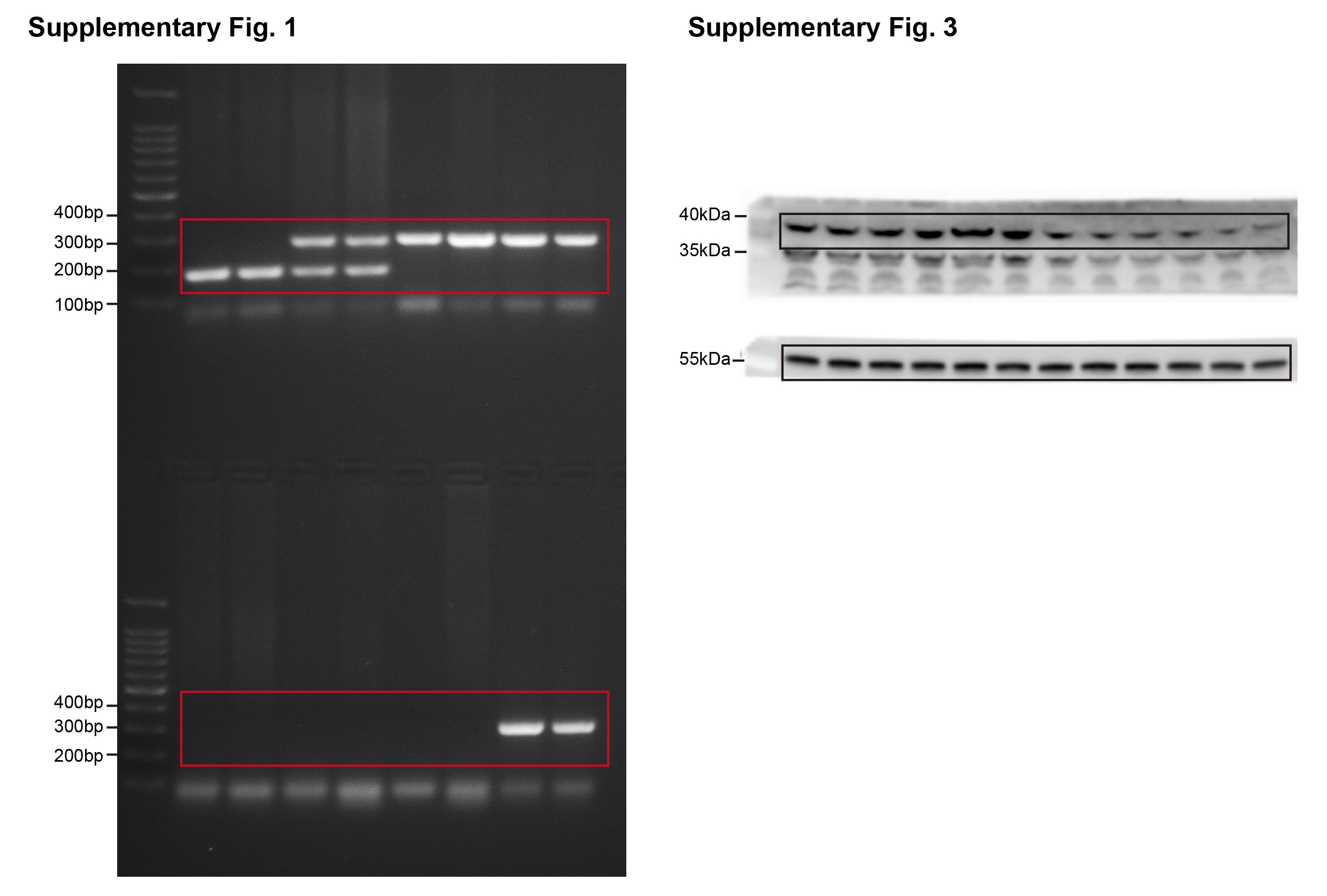

Supplement: Supplementary file 3 — Unprocessed gel and western blot images for the supplementary figures. [file 42255_2024_1036_MOESM3_ESM.jpg]

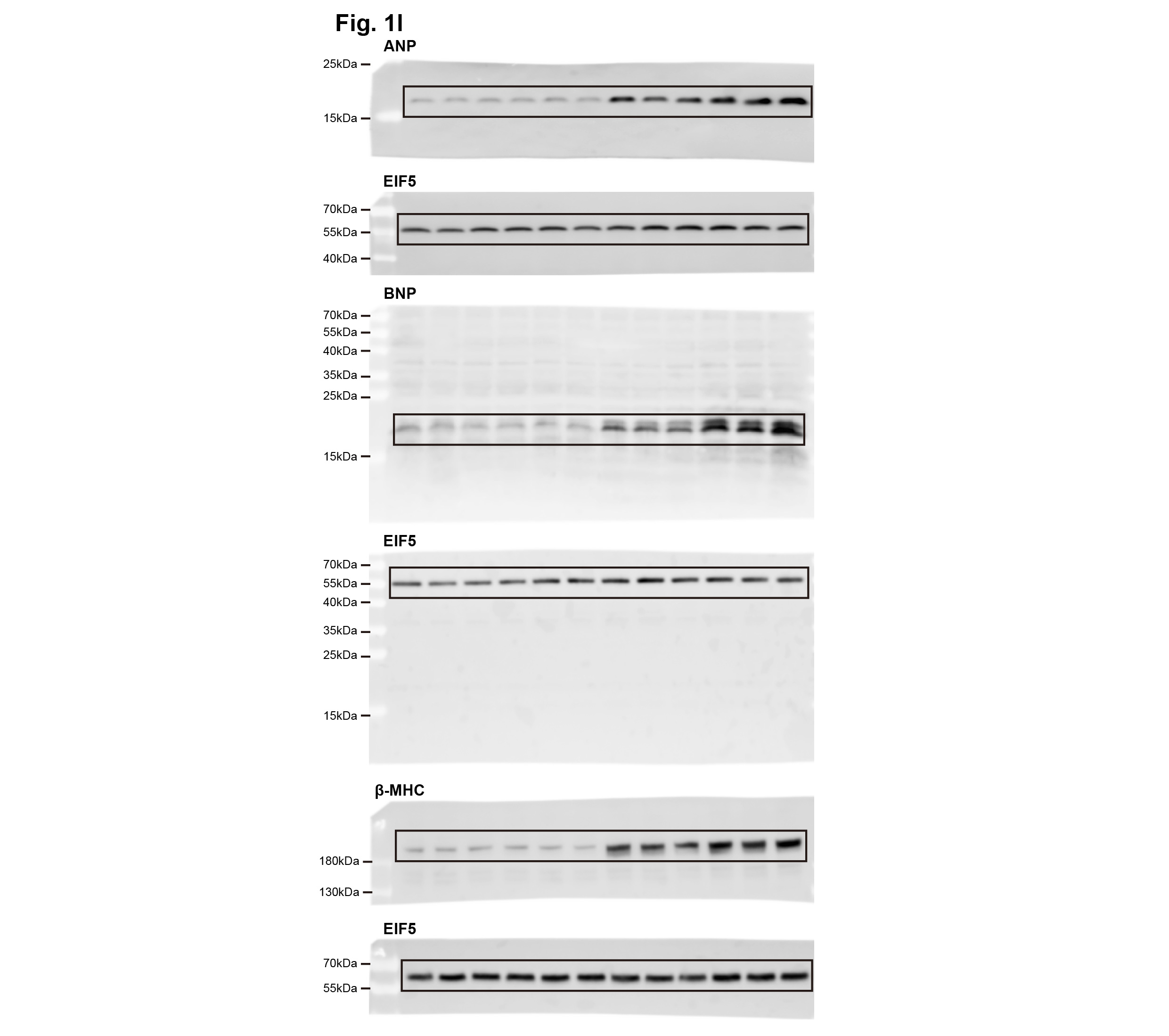

Supplement: Supplementary file 6 — Unprocessed western blots. [file 42255_2024_1036_MOESM6_ESM.jpg]

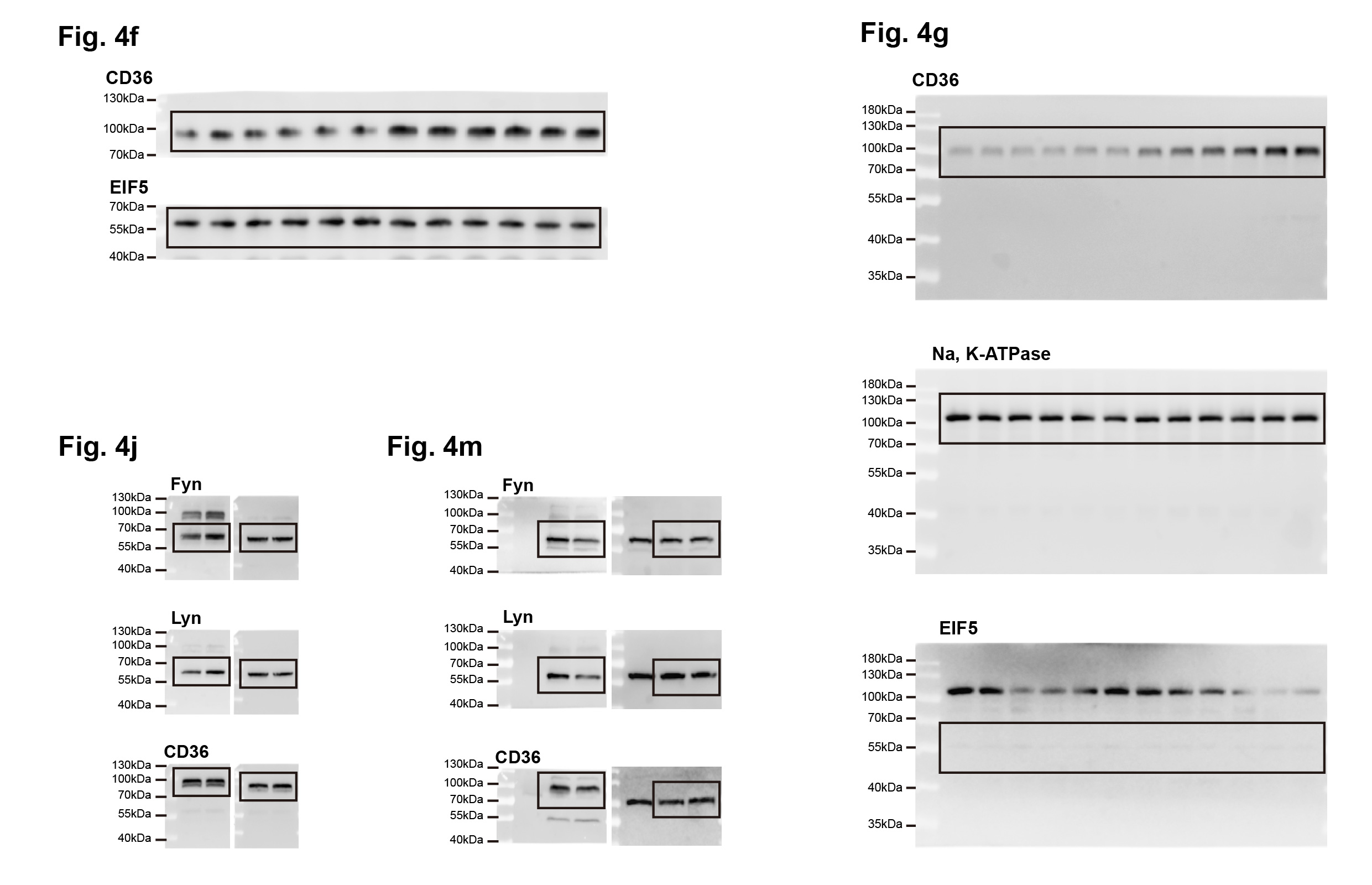

Supplement: Supplementary file 10 — Unprocessed western blots. [file 42255_2024_1036_MOESM10_ESM.jpg]

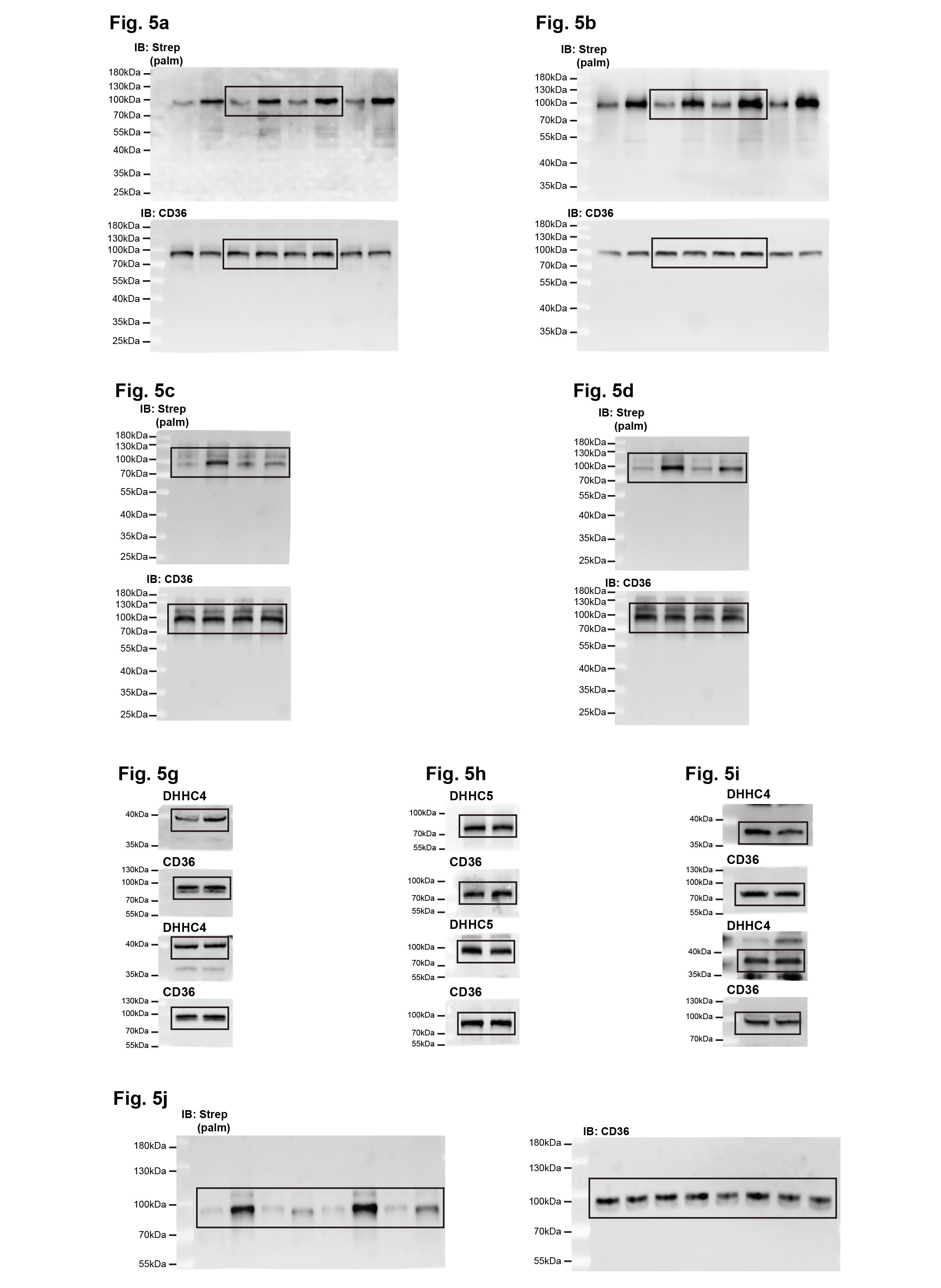

Supplement: Supplementary file 12 — Unprocessed western blots. [file 42255_2024_1036_MOESM12_ESM.jpg]

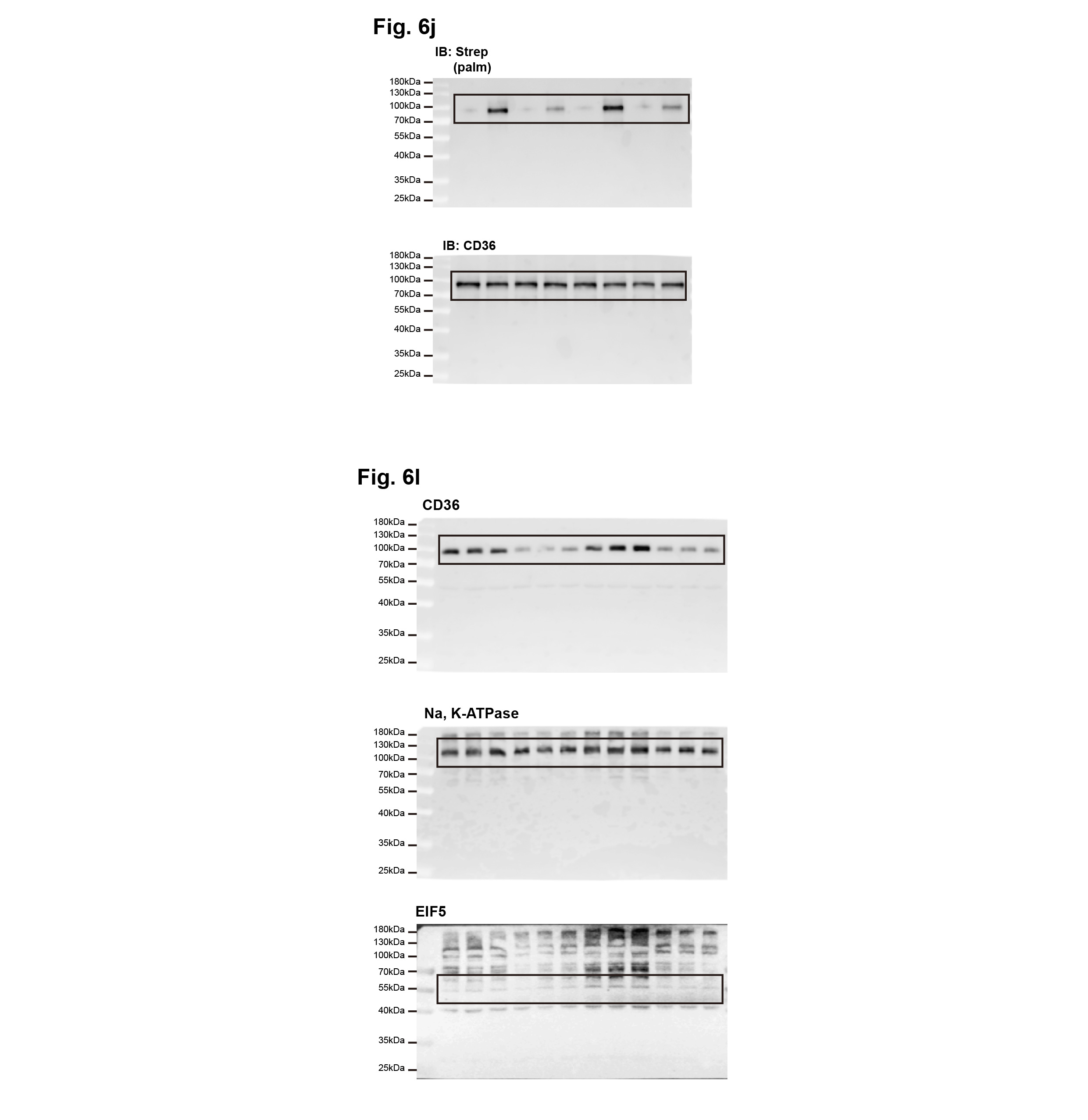

Supplement: Supplementary file 14 — Unprocessed western blots. [file 42255_2024_1036_MOESM14_ESM.jpg]

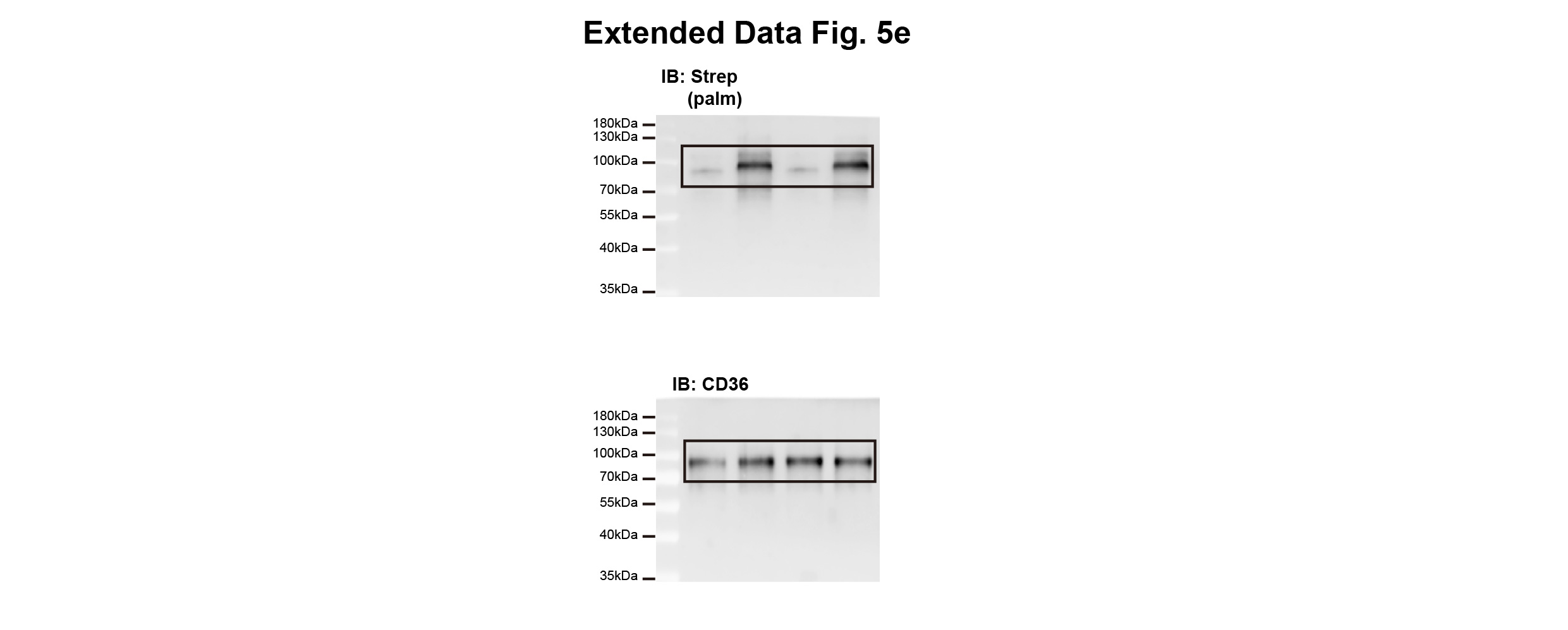

Supplement: Supplementary file 21 — Unprocessed western blots. [file 42255_2024_1036_MOESM21_ESM.jpg]

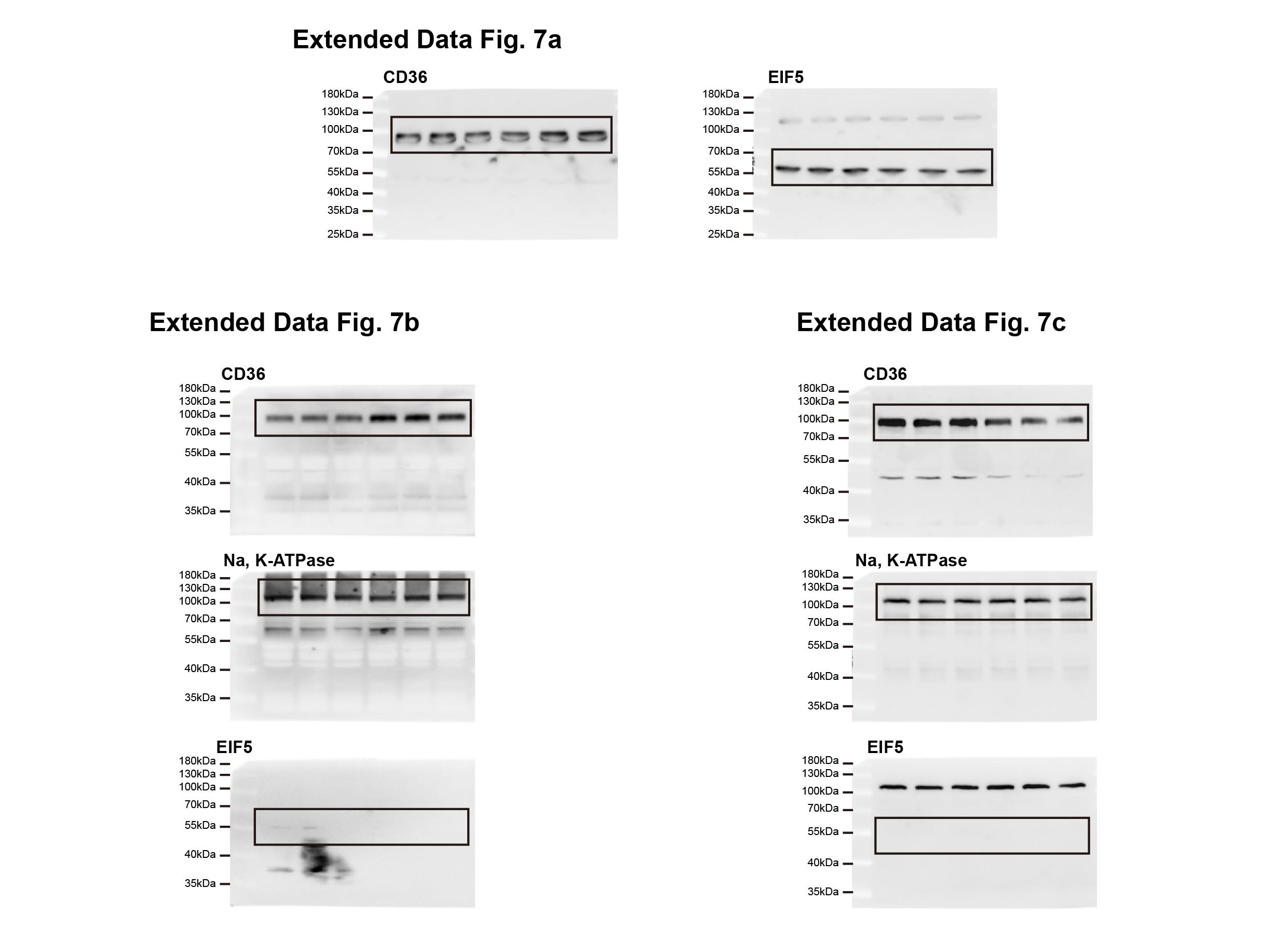

Supplement: Supplementary file 24 — Unprocessed western blots. [file 42255_2024_1036_MOESM24_ESM.jpg]

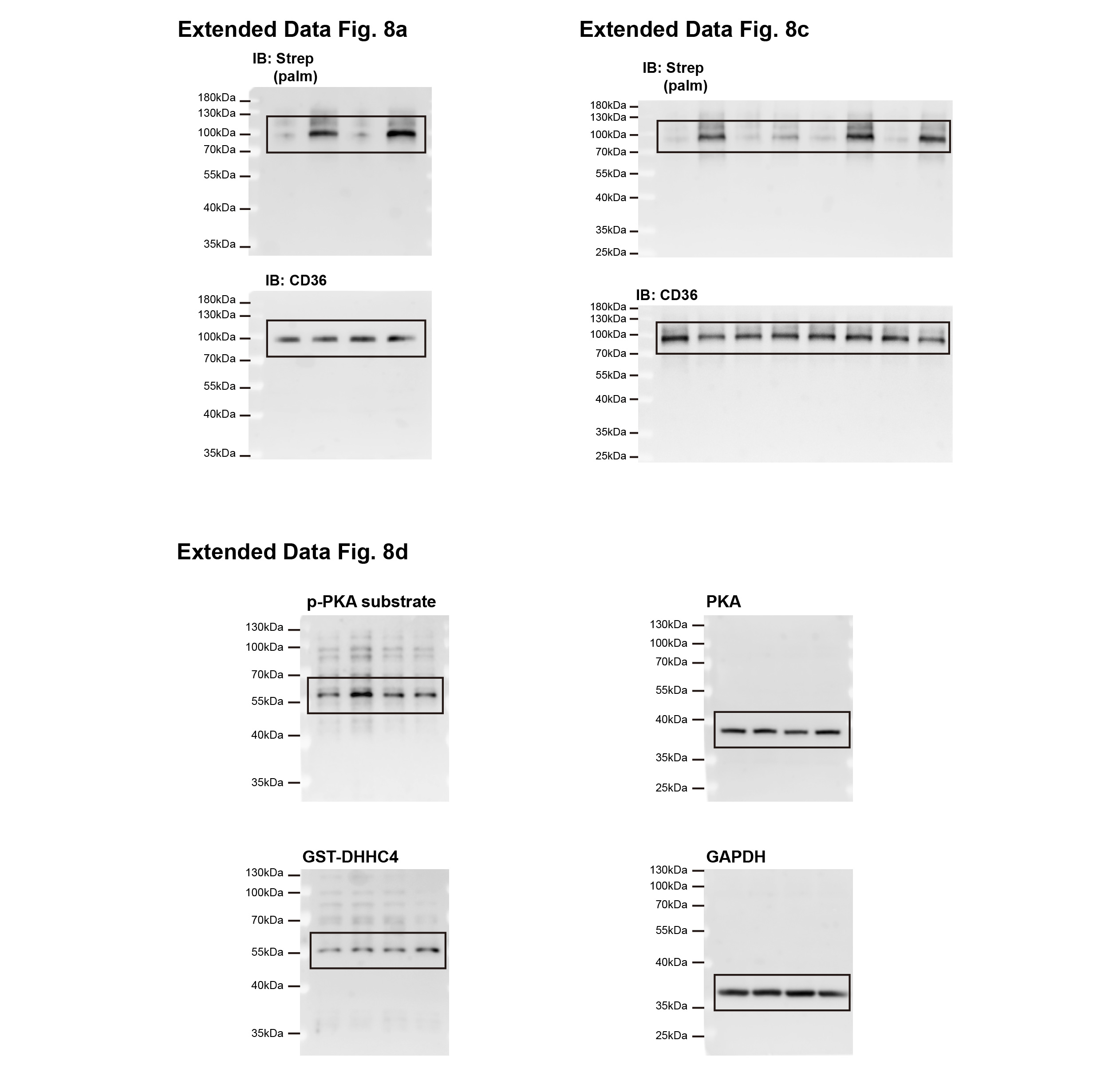

Supplement: Supplementary file 26 — Unprocessed western blots. [file 42255_2024_1036_MOESM26_ESM.jpg]
